# Machine learning based analyses on metabolic networks supports high-throughput knockout screens

**DOI:** 10.1186/1752-0509-2-67

**Published:** 2008-07-24

**Authors:** Kitiporn Plaimas, Jan-Phillip Mallm, Marcus Oswald, Fabian Svara, Victor Sourjik, Roland Eils, Rainer König

**Affiliations:** 1 Department of Bioinformatics and Functional Genomics, Institute of Pharmacy and Molecular Biotechnology, Bioquant, University of Heidelberg, Im Neuenheimer Feld 267, 69120 Heidelberg, Germany; 2 Division of Theoretical Bioinformatics, German Cancer Research Center (DKFZ), Im Neuenheimer Feld 280, 69120 Heidelberg, Germany; 3 Department of Discrete Optimization, Interdisciplinary Center for Scientific Computing, University of Heidelberg, Im Neuenheimer Feld 368, 69120 Heidelberg, Germany; 4 Center for Molecular Biology Heidelberg (ZMBH), Im Neuenheimer Feld 282, 69120 Heidelberg, Germany

## Abstract

**Background:**

Computational identification of new drug targets is a major goal of pharmaceutical bioinformatics.

**Results:**

This paper presents a machine learning strategy to study and validate essential enzymes of a metabolic network. Each single enzyme was characterized by its local network topology, gene homologies and co-expression, and flux balance analyses. A machine learning system was trained to distinguish between essential and non-essential reactions. It was validated by a comprehensive experimental dataset, which consists of the phenotypic outcomes from single knockout mutants of *Escherichia coli *(KEIO collection). We yielded very reliable results with high accuracy (93%) and precision (90%). We show that topologic, genomic and transcriptomic features describing the network are sufficient for defining the essentiality of a reaction. These features do not substantially depend on specific media conditions and enabled us to apply our approach also for less specific media conditions, like the lysogeny broth rich medium.

**Conclusion:**

Our analysis is feasible to validate experimental knockout data of high throughput screens, can be used to improve flux balance analyses and supports experimental knockout screens to define drug targets.

## Background

Defining drug targets and drug design is one of the major goals in biomedical research. In particular, metabolic enzymes have been successfully targeted by specific drugs to inhibit essential processes of pathogenic organisms in the human host [1]. Analyzing the metabolic network *in silico *helps to identify enzymes that are essential for the survival of the organism [2,3]. A general model for the metabolic network has been described by graph theoretical approaches and was applied to identify drug targets in pathogenic organisms [4]. The term 'damage' was used to assess enzymes that may serve as drug targets when their inhibition influences a substantial number of downstream metabolic reactions and products [5]. Furthermore, concepts of choke points and load points were successfully applied to estimate the essentiality of an enzyme [2,3]. Load points were defined as hot spots in the metabolic network (enzymes/metabolites) based on the ratio of the number of k-shortest paths passing through a metabolite/enzyme (in/out), and the number of nearest neighbor links (in/out) attached to it. This ratio was compared to the average load value in the network [2]. Choke points uniquely consume or produce a certain metabolite, which may make them indispensable. For example, in *Plasmodium falciparum *d-aminolevulinate dehydratase (ALAD) has been considered as a choke point [3] and was proven experimentally to serve as a valid antimalarial target [6].

Flux balance analyses (FBA) is a widely used and well established method to assess the essentiality of genes [7,8]. However, FBA approaches need clear definitions of nutrition availability and biomass production under specifically given environmental conditions (for a good overview of these aspects see e.g. [9]). High-throughput experiments have been performed to investigate the essentiality of a major portion or all genes in an organism [10-12]. For *Escherichia coli*, the essentiality of virtually all open reading frames was observed by a comprehensive knockout screen (KEIO collection [10]). This data enables to test the performance of an *in silico *metabolic model that predicts essential genes. Analyzing flux balances under aerobic glucose condition using the COBRA toolbox [13] and a newly reconstructed metabolic network of *E. coli *yielded 92% accuracy when predicting the essentiality of genes [8]. Feist and co-workers compared their predictions with the KEIO collection and yielded 88% for rich media conditions. In another study, FBA and the corresponding experimental knockout screen was performed to study the opportunistic behavior of the pathogen *Pseudomonas aeruginosa *with a systems view [14].

In this paper we propose an integrative machine learning approach applying a broad list of the described tools. The machine learning system was supplied with qualitative and quantitative descriptors derived from biochemical knowledge, genomic and transcriptomic data, and flux balance analyses. Using the KEIO collection [10] as the gold standard, we yielded an overall accuracy of 93% for rich media conditions. Comparative analysis between the flux balance approach and our machine learning approach yielded some improvements for FBA, namely to consider aminyl-tRNA reactions in modeling. Predictions that contradicted the KEIO collection were experimentally tested and successfully used to detect errors in the experimental data. Predicted reactions matching the experimental screen strengthen their candidacy as potential drug targets. Supporting this claim, 19 out of 37 predictions for novel targets were found in other literature with reported experimental evidence.

## Methods

### Network reconstruction

The data for the metabolic network was taken from a previous study and reconstructed in the same way (iAF1260, see Feist and co-workers [8]). Basically, the metabolic network was represented as an undirected bipartite graph consisting of metabolites and reactions as alternating nodes. This network was taken for our flux balance analyses. For all other analyses, unspecific compounds such as water, ATP, etc. were discarded.

### The gold standard

In order to demonstrate the efficiency of our approach we used data from the KEIO collection [10] as the gold standard. The dataset consisted of the phenotypic outcomes from a set of knockout mutants of single genes and was used to define the classes "essential" and "non-essential" for our reactions. Genes were knocked out by in-frame replacement of a PCR product containing a kanamycin resistance gene. The start-codon and the up-stream translational signal were not replaced and fully intact. After kanamycin treatment, in-frame single gene deletions were verified by PCR with loci specific primers. When they were unable to create a mutant that formed colonies on a plate, the mutated gene was considered to be essential. Knockout experiments were performed in LB rich medium and in glucose minimal medium, resulting in two datasets (denoted as rich medium and glucose minimal medium, respectively). For the rich medium, out of 4,288 tested genes, for 303 genes no mutants were found and therefore defined as being essential. Genes that were considered to be essential under rich medium condition were also considered as essential under glucose minimal medium condition. Additionally, to these genes, 119 genes were assigned to be essential in glucose minimal medium as they showed very slow growth in minimal media (growth rate ≤ 0.0926 in 24 hours). Experimental criteria for gene essentiality on glucose minimal medium are described in detail in [8,12]. Genes were mapped to the corresponding proteins, enzymes and reactions using the gene-protein-reaction Table from Feist *et al*. [8]. The reaction(s) associated with each gene were defined as essential or non-essential if there was no other way to activate the reaction(s) by other genes and if the coding gene was experimentally essential or non-essential, respectively. Otherwise they were discarded from our training and testing analysis. Furthermore, 133 reactions were discarded from the analysis, as the corresponding genes couldn't be defined. Finally, from 303 essential genes we determined a set of 231 essential and 1125 non-essential reactions under rich medium. Out of these 1125 non-essential reactions under rich medium, 107 reactions were defined as essential under glucose minimal medium. In total, 1356 reactions were used and the experimental results (KEIO) for their essentiality were taken as class labels of the reactions (samples) for training and validating the classifiers. Note that, we didn't use this experimental data for any features of the reactions.

### Defining the features

A list of relevant features was obtained from three different aspects: network topology, genomics and flux balance analyses. Table 1 gives an overview of all features and their abbreviations.

**Table 1:** List of all features

Short form	Explanation
	Topology features: local structures
RUP^a^	Reachable/Unreachable Products (RUP): more than or equal to one product cannot be produced when blocking a reaction
PUP	Percentage of Unreachable Products (PUP): the percentage of products which cannot be produced when blocking a reaction
NS^a^	Number of Substrates (NS)
NP^a^	Number of Products (NP)
NNR^a^	Number of Neighbouring Reactions (NNR)
NNNR^a^	Number of Neighbours of Neighbouring Reactions (NNNR)
CCV^a^	Clustering Coefficient Value (CCV): clustering coefficient of a reaction
DIR^a^	Directionality of a reaction (DIR)
	
	Topology features: deviations, choke points, load scores and damage
ND	Number of Deviations (ND)
APL	Average Path Length (APL): the average path length of the deviations
LSP^a^	Length of Shortest Path (LSP): the length of the shortest path of the deviations
CP	Choke Point (CP): a reaction is a choke point or not (Rahman *et al*, 2006)
LS^a^	Load Score (LS): load score of a reaction (Rahman *et al*, 2006)
NDR^a^	Number of Damaged Reactions (NDR): the number of damaged reactions after blocking a reaction (Lemke *et al*, 2004)
NDC^a^	Number of Damaged Compounds (NDC): the number of damaged compounds after blocking a reaction (Lemke *et al*, 2004)
NDRD^a^	Number of Damaged Reactions having no Deviations (NDRD): the number of damaged reactions that have no other alternative paths to be reached after blocking a reaction
NDCD^a^	Number of Damaged Compounds having no Deviations (NDCD): the number of damaged compounds that have no other alternative paths to be reached after blocking a reaction
NDCR^a^	Number of Damaged Choke point Reactions (NDCR): the number of damaged choke point reactions after blocking a reaction
NDCC^a^	Number of Damaged Choke point Compounds (NDCC): the number of damaged choke point compounds after blocking a reaction
NDCRD^a^	Number of Damaged Choke point Reactions having no Deviations (NDCRD): the number of damaged choke point reactions that have no other alternative paths to be reached after blocking a reaction
NDCCD^a^	Number of Damaged Choke point Compounds having no Deviations (NDCCD): the number of damaged choke point compounds that have no other alternative paths to be reached after blocking a reaction
	
	Gene expression data, genomic data and miscellaneous
NCG^a^	Number of Coding Genes (NCG): the number of coding genes for a reaction
H10^a^	Homology at 10^-10 ^(H10): the number of homologous genes with e-value cutoff 10^-10^
H7	Homology at 10^-7 ^(H7): the number of homologous genes with e-value cutoff 10^-7^
H5^a^	Homology at 10^-5 ^(H5): the number of homologous genes with e-value cutoff 10^-5^
H3^a^	Homology at 10^-3 ^(H3): the number of homologous genes with e-value cutoff 10^-3^
NRSG^a,b^	Number of Reactions from Same Genes (NRSG): the number of reactions derived from the same genes
NRSE^a,b^	Number of Reaction having Similar Expression (NRSE): the number of reactions that have similar expression (correlation coefficient >0.8)
MCC^a,b^	Maximum of Correlation Coefficients (MCC): maximum value of the correlation coefficients for all neighbouring reactions
	
	Flux distribution
BFV^a^	Biomass Flux Value (BFV): biomass flux value when blocking a reaction (under aerobic glucose condition)

^a^the optimized features, ^b^features from gene expression

### Topology based features

We set up a breadth first algorithm to investigate the network when a single reaction was blocked. We defined a reaction as essential for survival when basically the mutated network could not yield the products of the reaction from upstream substrates of the reaction. Hence, features were defined to describe if the knocked out reaction was substantial for producing its downstream metabolites or if these products could still be produced by other pathways. The investigation for each tested knocked out reaction was performed by the following algorithm.

i. All metabolites acting as input nodes (substrates) and output nodes (products) of the knocked out reaction were selected. The set of substrates S defined the input nodes and the set of products P defined the output nodes. To get a broader list of available substrates we integrated several other substrates into S. We included the substrates of the upstream reactions and the products of the downstream reactions into the sets S and P, respectively. Substrates of reactions that had at least one of the substrates S as a substrate was included into S. Further, substrates of reactions that had a metabolite out of P as a substrate were also included into S.

ii. Reactions were selected which used only available compounds as substrates.

iii. These selected reactions and their products were incorporated into the network. These products were set as new available metabolites in the network.

iv. Steps ii and iii were repeated until no further reactions could be identified for incorporation.

v. The output nodes that could be produced were counted (reachable products P).

After finishing the process, we used the number of defined output nodes that could be produced within the mutated network for two features, i.e. a quality feature defining if at least one product could not be produced (RUP, reachable/unreachable products), and the percentage of products that could not be produced (PUP, percentage of unreachable products).

We again run a breadth first search on the network to estimate possible deviations. This time we focused on relevant pathways by using the similarity measure from the SIMCOMP software [15]. SIMCOMP was used to define the most relevant substrates and products of each reaction. Starting from S, the breadth first search explored the network for finding the direct products of the knocked out reaction. When the algorithm visited these products, it stored the corresponding pathway and continued its search to find further alternative paths until the network was entirely explored or a maximal path length of 10 reactions was reached. We took the average path length (APL, average path length) and the shortest path length (LSP, length of shortest path) of the deviations as features for the classifier. The deviation features were used to find alternative pathways to produce products of the knocked out reaction by its substrates S. In the metabolic network, these substrates can also be consumed by other reactions yielding their products etc. Therefore, we kept track of alternative paths in the metabolic network for the potential of the organism to survive when a reaction was blocked. The organism may have many pathways to produce the products making the system more robust. Thus, we counted the number of possible alternative paths yielding feature ND (ND, number of deviations).

### Choke points, load points and damage

A reaction that uniquely consumes or produces a certain metabolite in the metabolic network is considered a choke point. Such a reaction shows high potential for essentiality [2,3]. We checked if an observed reaction was a choke point (CP, choke points). According to the concept of load scores from [2], we computed a load score of a reaction from the average number of pathways passing through the reaction, in comparison to the number of pathways for all metabolites in the network. We used the definition of damaged compounds/reactions reported by [5]. Basically, damage was defined by determining the potentially effected metabolites and reactions downstream of the knocked out reaction. We applied their definition for calculating the features NDR (NDR, number of damaged reactions) and NDC (number of damaged compounds). In turn, some damaged compounds/reactions might have been produced from alternative pathways. Therefore, we calculated the number of damaged compounds/reactions that did not have an alternative way to be reached from the substrates of the knocked out reaction (NDRD, number of damaged reactions having no deviations; NDCD, number of damaged compounds having no deviations). In addition to our analysis on damaged compounds/reactions, we also included the number of damaged choke points (NDCR, number of damaged choke point reactions; NDCC, number of damaged choke point compounds; NDCRD, number of damaged choke point reactions having no deviations; NDCCD, number of damaged choke point compounds having no deviations).

### Local topology features

The number of substrates and products of the knocked out reaction were counted (NS, number of substrates, and NP, number of products, respectively). Further, we defined features for the number of neighbouring reactions (NNR, number of neighbouring reactions), the number of neighbours of neighbouring reactions (NNNR, number of neighbours of neighbouring reactions) and the clustering coefficient (CCV, clustering coefficient values) [16,17] of the knocked out reaction. The reaction direction (DIR, directionality of a reaction) was taken from the model from Feist et al.[8].

### Gene expression data, genomic data and miscellaneous

For our case study, we collected gene expression data from a study observing the regulation during oxygen deprivation [18]. This dataset was taken to have a rather unspecific regulation, i.e. not of a small band but of a broad range of effected metabolic pathways. The gene expression data of each data-set was mapped onto the corresponding reactions. For a reaction that was catalysed by a complex of proteins, we took the mean of the gene expression values for the corresponding genes (for more details see [19]). Genes in the same pathway often show co-regulation [20]. Therefore, the maximum correlation coefficient of all neighboring reactions of the knocked out reaction (MCC, maximum correlation coefficient) and the number of reactions having similar gene expression (correlation coefficient > 0.8) were calculated (NRSE, number of reactions having similar expression). Together with the number of reactions coming from the same gene (NRSG, number of reactions from same genes), these features served the machine for estimating if the knocked out reaction was in a biosynthesis or degradation pathway. We also included the number of homologous genes that might have taken over the function of the knocked out gene. Homologous genes were searched using Blast [21] against all open reading frames of *E. coli *with four different e-value cutoffs, i.e. 10^-3^, 10^-5^, 10^-7^, and 10^-10 ^yielding the features H3, H5, H7 and H10, respectively. The method of the flux balance simulations is described in Results and discussion (section Comparing the performance to the performance of flux balance analyses).

### Machine learning

We applied Support Vector Machines from the R package e1071 [22] to classify between essential and non-essential reactions of the metabolic network. A radial basis function was used as the kernel function. Parameter optimization was performed for the regularization term that defined the costs for false classifications (5 steps for each, range: 2^n^, n = -4, -2, 0, 2, 4). The same range was taken for the kernel width γ. This optimization was realized by training with a grid search over all combinations of these parameters [22]. The sizes of the two classes differed significantly in our data set (essential: 17%, non-essential: 83%). For a broad spectrum of different precisions and sensitivities, we varied the weight factor for the positive instances from the data set with the optimized feature set in the range of 0.1 to 5.0. We performed a leave-one-out cross validation to measure the effectiveness of the machine learning method. A single reaction was selected as the validation data to be predicted and the remaining reactions as the training data. This was repeated for each reaction in the data set. For assessing the performance of the classifiers, we calculated the standard measures accuracy (number of correctly predicted reactions/number of all predicted reactions), sensitivity (number of true positives/(number of true positives + number of false negatives)), specificity (number of true negatives/(number of true negatives + number of false positives)), positive prediction value or precision (number of true positives/number of positively predicted reactions), negative prediction value (number of true negatives/number of negatively predicted reactions).

### Feature selection

The feature selection was done by a top-down approach. We trained the Support Vector Machines in terms of maximizing the overall accuracy using all features. Each single feature was discarded from the data set and the performance of the machine was observed. Testing the performance of the machine was done by a leave-one-out cross validation. The accuracies of the machines missing one feature were compared and the best machine kept for the next iteration. This was repeated until the accuracy did not increase. The machine with the best accuracy was selected as the best classifier and its features as the optimized feature set.

### Experimental protocol for the knockout verification

Knockout mutations were verified by PCR amplification of genomic loci expected to contain the 1327 base pair gene replacement cassette with specific primers (Table S4 in Additional file 1). Primers were chosen to have equal predicted melting temperatures of ~60°C and hybridised at specific distances upstream and downstream of the target gene. PCR reactions were performed directly from freshly grown bacterial colonies for 30 cycles at the annealing temperature of 54°C. The product sizes obtained from the KEIO collection strains were compared to those from the wild-type *E. coli *strain MG1655 on 1% agarose gels.

### Assembling a list of drug targets

To map enzymes with drug targets, drugs and their corresponding drug targets were selected from the drug database Drugbank [23]. We took drugs into account that affected any organism excepting humans and other mammals. Entries that were found as metabolites for a reaction in the KEGG database [24] were discarded to restrict our drug list to non-endogenous compounds. The targets' annotated EC numbers of the remaining drugs were collected as our validated drug targets.

## Results and discussion

**Figure 1:**
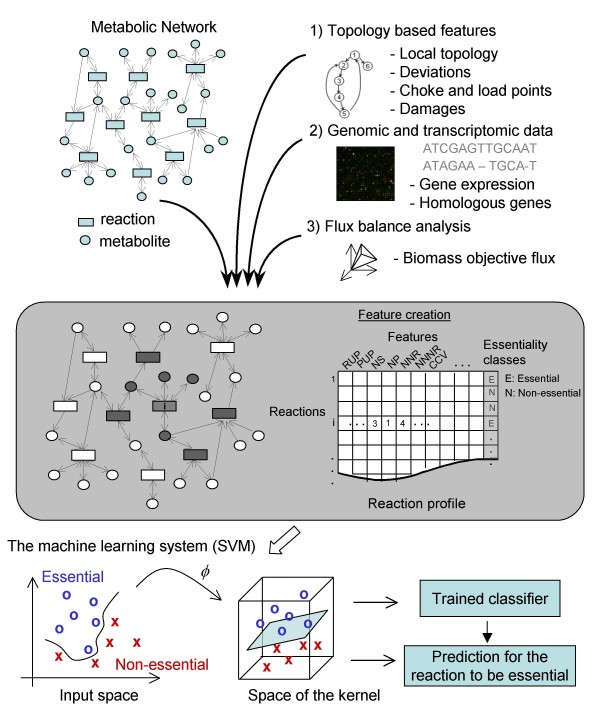
**Schematic overview of the method**. For each reaction, features were calculated describing 1) its local topology in the metabolic network, 2) its genomic and transcriptomic relations, and 3) biomass production using flux balance analyses when discarding the reaction. E.g. if a given reaction *i *had three educts, one product and 4 neighbors, the feature NS (number of substrates), NP (number of products), and NNR (number of neighboring reactions) were set to 3, 1 and 4, respectively. A table for the reaction profiles was created and used for training the classifier to distinguish between essential and non-essential reactions. A Support Vector Machine (SVM) was applied as a classifier to find the optimal separating hyperplane.

An overview of the machine learning procedure is shown in Figure 1. The machine learning system was trained and validated with a large set of features. Firstly, local topology based features where used to qualitatively describe possible flux deviations. Secondly, choke and load points were defined and damage was used to describe the qualitative flux load and down stream effects of the knocked down reaction. Thirdly, functional genomics data, such as co-expression of genes for up- and down-stream reactions was used to indicate conjoint reactions in a pathway. In addition to these features, we considered the existence of homologous genes for the corresponding knockout reaction, which may be expected to take over the function of the knocked out gene. Finally, we used flux balance analyses to estimate the network's biomass production following knocking out a reaction. A list of all features is given in Table 1.

### Performance of the machine learning algorithm

Due to the small data set of 1356 reactions for training and validation, we performed a leave-one-out cross validation to measure the effectiveness of our machine learning system. Using the KEIO-collection data of rich medium as the reference, we gained an overall accuracy of 92% when all features were taken (Table 2). To increase the performance, we did a systematic feature reduction within a top-down procedure. We yielded a better result with an optimized feature set of 25 features (accuracy = 93%, see Table 2). These 25 features may be regarded as the dominating factors for leading to a good performance. To find out which of them are more relevant, we again started the top down procedure, now stopping at the first step. For all features, we compared the accuracy for each classifier lacking of one feature, respectively. It turned out that loosing the feature NNNR yielded the worst classification performance (accuracy -0.89 compared to the classifier with all features) and therefore hinting for being the most relevant feature. This feature was followed by NRSE (-0.82), BFV (-0.74), NNR (-0.52) and H10 (-0.52). Interestingly, these first five features span already the whole set of our feature categories (NNNR and NNR: network toplogy; NRSE: gene expression, genomics; H10: homology, genomics; and BFV: flux balance analysis).

**Table 2:** Performance of machine learning based predictions on rich media condition

	Machine Learning	Machine Learning
	(all 30 features)	(25 optimized features)
true positives	168	174
true negatives	1078	1092
false positives	47	33
false negatives	63	57
sensitivity (recall)	72.73%	75.32%
specificity	95.82%	97.07%
positive predictive values (precision)	78.14%	84.06%
negative predictive values	94.48%	95.04%
overall accuracy	91.89%	93.36%

**Figure 2:**
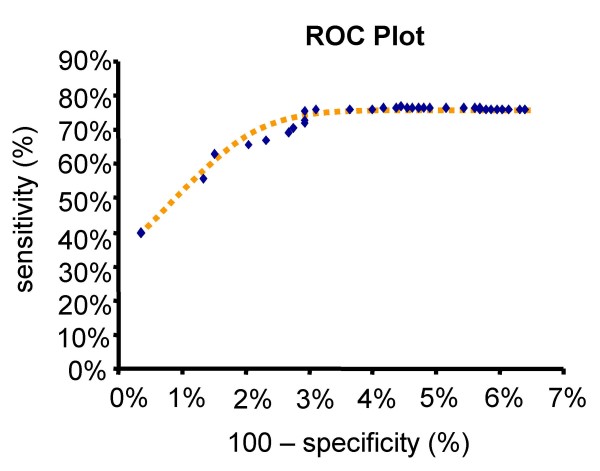
**ROC-curve showing our prediction results with different weight factors for positive instances**. Each (blue) diamond shows the result for a different weight. From left to right, the weight was increased from 0.1 to 5.0, by a step size of 0.1. When the weight factors were higher than 1.0, the sensitivity remained constant. The dotted line was manually fitted.

The sizes of the two classes "essential reactions" and "non-essential reactions" differed significantly in our data set (essential: 17%, non-essential: 83%). For obtaining different stringencies, we weighted the positive instances by a factor ranging from 0.1 to 5.0 with a step size of 0.1 (Figure 2). The sensitivity increased significantly from smaller to higher weights, reaching a plateau for weight factors of 1.0 or more. As expected, with a smaller weight the classifier tended to be overwhelmed by the large negative class. More positive instances were recognized when their weight factor increased. The highest specificity (99%) and the best precision (95%) was yielded by the first data point with a weight factor of 0.1. This is beneficial when predicting drug targets with high reliability. Alternatively, to avoid overlooking potential targets, increased sensitivity can be achieved by raising weight factor to at least 1.0 (sensitivity = 75%). In the following, all analyses were performed with a weight factor of one.

### Identifying drug targets

**Figure 3:**
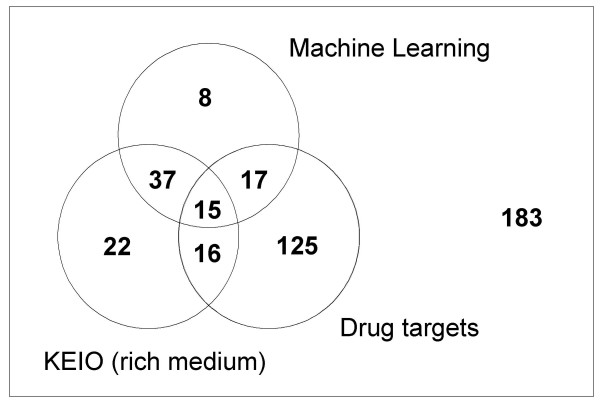
**The investigated enzymes**. Three sets are shown: essential enzymes found by the machine learning approach (top circle), essential enzymes found by the KEIO knockout screen [10] (bottom-left circle) and enzymes that are valid drug targets taken from the database Drugbank [23] (bottom-right).

We compared the enzymes of our predictions and the results from the KEIO collection to a comprehensive list of valid drug targets from the Drugbank database [23] (Figure 3). Surprisingly, 80% of the drug target enzymes were neither found by the KEIO high-throughput screen nor by our machine. This may be due to the fact that these drug targets are for a broad range of organisms having different topologies of the metabolic networks and may have different alternative pathways for the corresponding drug targets. It should be noted that this study focused on reactions being essential under rich media conditions. We suggest 37 promising drug target enzymes that are not in the Drugbank database, which are validated by the intersection of our predictions with the results from the experimental KEIO screen. A list of these reactions with references for reported experimental evidences is given in Table S1 [in Additional file 1].

### Comparing the performance of the machine learning approach to flux balance analyses

We performed a single reaction deletion on the network and calculated flux values by FBA using the Cobra Toolbox [13] to assess essential reactions under aerobic glucose minimal media conditions (as described in the supplementary material of Feist et al. 2007). In this analysis, a reaction was assessed to be essential if the respective prediction of the mutated network's maximal biomass production was < 1% of the wildtype's biomass production. The biomass objective function used in the analysis was also taken as explained in [8]. Note that simulating rich media conditions is challenging, as it is difficult to characterize the uptake rates for each compound of a rich medium (Adam Feist, personal communication 2008). Because of this, we compared the performances of our approach with the FBA on glucose minimal media. 338 reactions were found to be essential in glucose minimal medium according to the experimental criteria for gene essentiality under glucose minimal media in the KEIO collection [8,10,12]. 996 reactions were identified as non-essential. The remaining reactions had no associated gene, were exchange reactions, or could not clearly be identified. The FBA approach detected the essentiality of a reaction under aerobic glucose minimal condition with an accuracy of 86%, a sensitivity of 52% and a specificity of 98%. We performed our machine learning under glucose minimal conditions with and without BFV (Biomass flux value from FBA simulation) and found BFV to improve the results (Table 3). With BFV, our approach yielded 90% precision and 79% recall of experimental results, compared with FBA results of 87% precision and 51% recall. Results for all reactions are given in Table S2 [Additional file 2].

**Table 3:** Comparison of our machine learning method and Flux Balance Analyses on glucose minimal media condition

Performance	ML\BFV^1^	ML^2^	FBA^3^
true positives	192	266	174
true negatives	932	968	971
false positives	64	28	25
false negatives	146	72	164
sensitivity	56.80%	78.70%	51.48%
specificity	93.57%	97.19%	97.49%
positive predictive values	75.00%	90.48%	87.44%
negative predictive values	86.46%	93.08%	85.55%
overall accuracy	84.26%	92.50%	85.83%

^1^machine learning without the feature BFV (biomass flux value from the FBA).

^2^machine learning including the feature BFV

^3^Flux Balance Analysis

### Improving flux balance simulations

**Figure 4:**
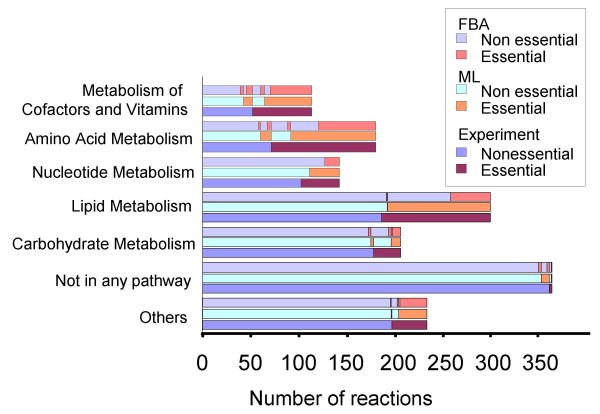
**Comparison of our machine learning predictions, FBA and the experimental data, according to different pathways**. For each pathway of KEGG [24], the lowest bars represent the experimental result (KEIO), reactions are grouped into non-essential (left, blue) and essential (right, magenta). The mid and top bars show the prediction results of our machine learning approach and flux balance analyses, respectively. Larger differences between the machine learning prediction and the FBA were in the amino acid metabolism and lipid metabolism.

Figure 4 shows a comparison of our method and FBA by categorizing the results according to the KEGG pathways [24]. The essential reactions that were found by our machine learning approach but not by the FBA were mostly reactions in amino acid metabolism and lipid metabolism. In amino acid metabolism, the tRNA transferases of almost all amino acids were found to be essential by the machine learning approach but not by FBA. We improved FBA simulations by adding the corresponding aminyl-tRNA reactions and their products to the biomass objective function. This ensured that all tRNA transferases would be predicted as essential by the FBA method. The simulations subsequently gained better results by correctly predicting the essentiality of the aminyl-tRNA reactions.

### Using our approach as a means to validate the experimental knockout screen

Predicting a different outcome from experimental high throughput screen (KEIO) may be due to either an error in our algorithm, *or *an error within the experimental knockout screen. We examined our lists of false positives and false negatives by two experimental set-ups. Our list of false negatives contained 71 genes which our algorithm predicted to be non-essential under glucose minimal condition in contradiction to the outcome of the KEIO experiment [10]. For 33 of them we obtained corresponding knockout clones from the KEIO library (growing on rich media), and grew them on M9 glucose medium. Indeed, we were able to grow 9 out of 33 clones with good growth rates (OD600 ≥ 0.2 after 48 hours) and 3 clones with reasonable growth rates (OD600 between 0.07 and 0.2 after 48 hours). The complete list is given in Table S3 [in Additional file 1]. In turn, we also tested the list of false positives, for which our algorithm predicted 33 genes to be essential, in contrast to the experimental high throughput screen. We assumed that some of these genes weren't knocked out correctly. Baba et al. (2006) provided a validity estimation for their clones. We compared our results to their estimations and selected 6 genes, for which mutants they estimated to be less than or equal to 37.5% correct. For 5 out of these 6 genes (alaS, coaA, coaE, glyS and hemE) PCR with specific primer pairs (Table S4 in Additional file 1) yielded two products with sizes corresponding to wild-type and knockout alleles, respectively. This indicated that the genes were not correctly knocked out and the wild-type gene was still present. No PCR product was observed for the ileS knockout. Additionally we tested another 4 genes out of our list, for which mutations were stated to be 100% correct by Baba et al. Indeed, for all of those genes (aspC, epd, luxS, thiE) only the correct PCR product corresponding to the knockout allele was observed.

## Conclusion

Defining drug targets is a challenging task. Many experiments rely on a conditional essentiality screen of genes to define the associated enzymes as possible drug targets. Machine learning methods can help to validate this experimental data. Our approach used the experimental knockout data for *E. coli *from KEIO [10]. The machine was trained with this data and predicted quite accurately the experimental outcomes. Most methods based on graphical networks aim at finding out weak points in the network. We set up a machine learning system that integrates features describing the network topology and functional genomics properties in an elaborated way. By this we gained two valuable insights. Firstly, we could see that the topologic, genomic and transcriptomic data describing the network attributes was sufficient for defining the essentiality of a certain reaction. For pathogens it is often hard to define the environmental parameters which are complex and changeable as e.g. for intestinal infections. Our approach can, in principle, handle all media conditions, as shown for rich media conditions in this study. Rich media conditions may better reflect the situation of the pathogens in the host (like e.g. in the gut), in comparison to minimal media conditions with clearly defined carbon sources for which flux balance analyses can be well adapted. A second benefit of our study is the experimental validation and support for estimations of potential drug targets. When regarding the intersection of our results and the KEIO collection, we found 37 potential targets for novel drugs, for 19 out of which we could find some reported experimental evidence in the literature. An advantage of machine learning approaches is to easily change the stringency parameter, e.g. for increasing precision to avoid loosing potential candidates, the weight factor for the positive instances can be increased. We used gene expression data from *E. coli *wild-type and single knock out strains. The single knock outs were regulators for respiration effecting a large number of genes and also the treatment was rather unspecific (growth in oxygen rich and deprived conditions). Hence, a large portion of network pathways of the metabolic network was differentially expressed [25]. Within the presented approach, data of such pathway unspecific examinations suited well to let the classifier learn which neighboring enzymes jointly work together. Therefore, also multiple gene co-expression datasets for a variety of conditions may suit well for our approach. However, it needs still to be investigated which gene expression data suits best to optimize the performance.

We have presented a system that could be broadly applied to systems seeking potential drug targets for a variety of substantial bacterial infections and other organisms. For *E. coli *we benefited from a rich data pool including a well elaborated metabolic network, a genome wide knock out viability screen, the genome sequence and a feasible gene expression dataset. Nowadays, the genomic sequence may not be the limiting factor for most applications as a remarkable number of genomes has been sequenced or will be sequenced in the next future. As our approach uses unspecific gene expression data also this can be obtained from publically available resources or obtained by rather straightforward experiments. Very well elaborated metabolic networks have been assembled for some organisms (e.g. *B. subtilis *[11]*, H. pylori *[26]*, M. barkeri *[27]*, M. tuberculosis *[28]*, S. cerevisiae *[29]) to which we expect that our method can be transferred without major difficulties. Further networks can be received for a large amount of organisms from existing excellent databases like BioCyc [30] and Kegg [24]. It will be challenging to exploit these networks with our method. Finally, until now, for our approach the genome wide essentiality screen is still substantial and laborious. A methodological very challenging task remains to employ our approach across different organisms, by e.g. using the essentiality screen of one organism to infer the information to another.

## Authors' contributions

KP, J–PM, RE and RK put up the general concept and design of the study. KP carried out the data analysis. MO and KP conceptualized and implemented the breath first search algorithm. FS and VS validated the model predictions experimentally in the lab. KP and RK drafted the manuscript. All authors read and approved the final manuscript.

## Supplementary Material

Additional File 1 Supplementary tables. Tables S1, S3 and S4. Click here for file

Additional File 2 Supplementary Table S2. Results of all Reactions. Click here for file
