# Supplementary material for: Machine learning based analyses on metabolic networks supports high-throughput knockout screens
Source: BMC Syst Biol. 2008 Jul 24;2:67. doi: 10.1186/1752-0509-2-67 (PMC2526078; doi:10.1186/1752-0509-2-67)
Supplement: Additional File 1 — Supplementary tables. Tables S1, S3 and S4. [file 1752-0509-2-67-S1.pdf]

Content of this file:

Supplementary Table S1. List of potential drug targets

Supplementary Table S3. Results from our growth experiments

Supplementary Table S4. List of the applied primer pairs and our experimental results of testing for correctly knocked out genes

Supplementary Table S1. List of potential drug targets

EC	ORF	Gene symbol	Evidence from the literature	Reference
1.1.1.158	b3972	murB	In *B. antracis*, antisense dependent MurB2 expression gave synergistic response to beta-lactam antibiotics	(Kedar *et al*, 2007)
1.3.1.10	b1288	fabI	FabI is a well known drug target against micro-organisms including *E. coli*	(Kitagawa *et al*, 2007)
2.3.1.15	b4041	plsB	Mutations in plsB occur in *E. coli* strains with multi-drug tolerance.	(Spoering *et al*, 2006)
2.3.1.51	b3018	plsC	nothing found	
2.7.1.130	b0915	lpxK	It was shown that growth of *E. coli* is inhibited when *lpxK* is inactivated.	(Garrett *et al*, 1998)
2.7.1.26	b0025	ribF	nothing found	
2.7.4.8	b3648	gmk	*Salmonella* with *gmk* mutations showed growth dependence on adenine.	(Beck *et al*, 2003)
2.7.7.18	b0639	nadD	nothing found	
2.7.7.2	b0025	ribF	nothing found	
2.7.7.3	b3634	coaD	nothing found	
2.7.7.41	b0175	cdsA	nothind found	
2.7.8.13	b0087	mraY	MraY inhibitors serve as novel antibacterial agents.	(Dini, 2005)
2.7.8.5	b1912	pgsA	*PgsA* codes for an essential enzyme of *Mycobacterium smegmatis* that shows promise as a drug target for anti-tubercolosis therapy	(Jackson *et al*, 2000)
2.7.8.8	b2585	pssA	nothing found	
3.5.1.18	b2472	dapE	*Heliobacter* strains lacking *dapE* were dependent on diaminopimelic acid.	(Karita *et al*, 1997)
3.5.4.16	b2153	folE	nothing found	
3.5.4.26	b0414	ribD	nothing found	
3.5.4.9	b0529	folD	nothing found	
4.1.1.65	b4160	psd	*Psd* null mutants of *E. coli* were non-motile.	(Karita *et al*, 1997)
4.1.2.16	b1215	kdsA	*E. coli* containing missense mutations in *kdsA* stopped dividing and cell growth.	(Fujishima *et al*, 2002)
4.2.1.52	b2478	dapA	Dihydrodipicolinate synthase (EC 4.2.1.52) is essential for lysine biosynthesis in *E. coli.*	(Vauterin *et al*, 2000)
4.3.1.8	b3805	hemC	nothing found	
4.3.2.2	b1131	purB	*PurB* mutants of *Lotus japonicus* exhibit purine auxothrophy.	(Okazaki *et al*, 2007)
6.1.1.11	b0893	serS	nothing found	

6.1.1.12	b1866	aspS	nothing found	
6.1.1.16	b0526	cysS	Cysteinyl-tRNA synthetase is essential for protein synthesis	(Bunjun *et al*, 2000)
6.1.1.17	b2400	gltX	nothing found	
6.1.1.18	b0680	glnS	nothing found	
6.1.1.19	b1876	argS	Mutations of *argS* and *leuS* are found in *E. coli* strains which are resistant to the antibiotic novobiocin.	(Jovanovic *et al*, 1999)
6.1.1.2	b3384	trpS	nothing found	
6.1.1.20	b1713,	pheT,	nothing found	
	b1714	pheS		
6.1.1.21	b2514	hisS	Mutants expressing a structurally altered HisS protein require external histidine.	(Straus and Ames, 1973)
6.1.1.22	b0930	asnS	AsnS inhibitors are used as anticancer drugs.	(Richards and Kilberg, 2006)
6.1.1.4	b0642	leuS	Mutations of *argS* and *leuS* are found in *E. coli* strains which are resistant to the antibiotic novobiocin.	(Jovanovic *et al*, 1999)
6.1.1.9	b4258	valS	nothing found	
6.3.2.13	b0085	murE	S. aureus strains which are resistant against the antibiotic methicillin show mutations in murE which is needed for cell wall synthesis.	(De Lencastre *et al*, 1999)
6.3.2.15	b0086	murF	4-phenylpiperidine was reported to inhibit the MurF enzyme and may contribute to antibacterial acivity by interfering with cell wall biosynthesis.	(Baum *et al*, 2007)

References for Supplementary Table S1

Baum EZ, Crespo-Carbone SM, Klinger A, Foleno BD, Turchi I, Macielag M, Bush K (2007) A MurF inhibitor that disrupts cell wall biosynthesis in Escherichia coli. *Antimicrobial agents and chemotherapy* **51:** 4420-4426.

Beck BJ, Huelsmeyer M, Paul S, Downs DM (2003) A mutation in the essential gene gmk (encoding guanlyate kinase) generates a requirement for adenine at low temperature in Salmonella enterica. *Journal of bacteriology* **185:** 6732-6735.

Bunjun S, Stathopoulos C, Graham D, Min B, Kitabatake M, Wang AL, Wang CC, Vivares CP, Weiss LM, Soll D (2000) A dual-specificity aminoacyl-tRNA synthetase in the deep-rooted eukaryote Giardia lamblia. *Proceedings of the National Academy of Sciences of the United States of America* **97:** 12997-13002.

De Lencastre H, Wu SW, Pinho MG, Ludovice AM, Filipe S, Gardete S, Sobral R, Gill S, Chung M, Tomasz A (1999) Antibiotic resistance as a stress response: complete sequencing of a large number of chromosomal loci in Staphylococcus aureus strain COL that impact on the expression of resistance to methicillin. *Microbial drug resistance (Larchmont, NY* **5:** 163-175.

Dini C (2005) MraY Inhibitors as Novel Antibacterial Agents. *Current topics in medicinal chemistry* **5:** 1221-1236.

Fujishima H, Nishimura A, Wachi M, Takagi H, Hirasawa T, Teraoka H, Nishimori K, Kawabata T, Nishikawa K, Nagai K (2002) kdsA mutations affect FtsZ-ring formation in Escherichia coli K-12. *Microbiology (Reading, England)* **148:** 103-112.

Garrett TA, Que NL, Raetz CR (1998) Accumulation of a lipid A precursor lacking the 4'-phosphate following inactivation of the Escherichia coli lpxK gene. *The Journal of biological chemistry* **273:** 12457-12465.

Jackson M, Crick DC, Brennan PJ (2000) Phosphatidylinositol is an essential phospholipid of mycobacteria. *The Journal of biological chemistry* **275:** 30092-30099.

Jovanovic M, Lilic M, Janjusevic R, Jovanovic G, Savic DJ (1999) tRNA synthetase mutants of Escherichia coli K-12 are resistant to the gyrase inhibitor novobiocin. *Journal of bacteriology* **181:** 2979-2983.

Karita M, Etterbeek ML, Forsyth MH, Tummuru MK, Blaser MJ (1997) Characterization of Helicobacter pylori dapE and construction of a conditionally lethal dapE mutant. *Infection and immunity* **65:** 4158-4164.

Kedar GC, Brown-Driver V, Reyes DR, Hilgers MT, Stidham MA, Shaw KJ, Finn J, Haselbeck RJ (2007) Evaluation of the metS and murB loci for antibiotic discovery using targeted antisense RNA expression analysis in Bacillus anthracis. *Antimicrobial agents and chemotherapy* **51:** 1708-1718.

Kitagawa H, Kumura K, Takahata S, Iida M, Atsumi K (2007) 4-Pyridone derivatives as new inhibitors of bacterial enoyl-ACP reductase FabI. *Bioorganic & medicinal chemistry* **15:** 1106-1116.

Okazaki S, Hattori Y, Saeki K (2007) The Mesorhizobium loti purB gene is involved in infection thread formation and nodule development in Lotus japonicus. *Journal of bacteriology* **189:** 8347-8352.

Richards NG, Kilberg MS (2006) Asparagine Synthetase Chemotherapy. *Annu Rev Biochem.*

Spoering AL, Vulic M, Lewis K (2006) GlpD and PlsB participate in persister cell formation in Escherichia coli. *Journal of bacteriology* **188:** 5136-5144.

Straus DS, Ames BN (1973) Histidyl-transfer ribonucleic acid synthetase mutants requiring a high internal pool of histidine for growth. *Journal of bacteriology* **115:** 188-197.

Vauterin M, Frankard V, Jacobs M (2000) Functional rescue of a bacterial dapA auxotroph with a plant cDNA library selects for mutant clones encoding a feedback-insensitive dihydrodipicolinate synthase. *Plant J* **21:** 239-248.

Supplementary Table S3. Results from our growth experiments

Shown are the columns running number, ORF-id, gene symbol, our experimental results, classified according to OD600 (minus: < 0.07, no growth; plus/minus: between 0.07 and 0.2, slow growth; plus: > 0.2: growth), our OD600 after 48 hours (measured in duplicate), and OD600 in glucose MOPS medium with 2nM Pi conditions after 24 hours as given by Baba et al. (2006).

#	ORFs	Gene	Our experimental result	our OD600 (48 hours)	OD600 of Baba et a.l (24 hours)
1	b1638	pdxH	-	0.001	0.005
2	b3870	glnA	-	0.000	0.005
3	b2913	serA	-	0.002	0.007
4	b2508	guaB	-	0.005	0.005
5	b3772	ilvA	-	0.001	0.014
6	b2551	glyA	-	0.000	0.002
7	b1136	icd	-	0.003	0.029
8	b0002	thrA	-	0.029	0.023
9	b0032	carA	+	0.432	0.003
10	b3008	metC	+/-	0.152	0.029
11	b0720	gltA	-	0.032	0.014
12	b1264	trpE	-	0.016	0.02
13	b0033	carB	-	0.029	0
14	b2530	iscS	+/-	0.084	0.028
15	b2416	ptsI	-	0.039	0.018

16	b0115	aceF	+/-	0.070	0.091
17	b0116	lpd	-	0.000	0.061
18	b0242	proB	-	0.045	0.003
19	b2415	ptsH	+	0.747	0.066
20	b3829	metE	-	0.008	0.008
21	b3916	pfkA	-	0.018	0.087
22	b4388	serB	-	0.019	0.009
23	b0243	proA	-	0.006	0.007
24	b0775	bioB	+	0.959	0.049
25	b0776	bioF	+	0.795	0.025
26	b0778	bioD	+	1.15	0.037
27	b3731	atpC	+	0.833	0.038
28	b3737	atpE	+	0.671	0.016
29	b1260	trpA	-	0.029	0.008
30	b1261	trpB	-	0.003	0.008
31	b3281	aroE	-	0.001	0.007
32	b3738	atpB	+	0.989	0.014
33	b3940	metL	-	0.000	0.011
34	wildtype		+	0.702	

Supplementary Table S4. List of the applied primer pairs and our experimental results of testing for correctly knocked out genes

#	ORFs	Gene	%correct	Experimental result	fwd primer	rev primer
1	b0103	coaE	25%	2 PCR-bands	5'-aagggtaagagcgcaactcc-3'	5'-tggcaatccaggtttctacc-3'
2	b2697	alaS	25%	2 PCR-bands	5'-ccgactgaacgcatacgg-3'	5'-tacctggttgccctttaccc-3'
3	b3559	glyS	25%	2 PCR-bands	5'-acattcagggcgtagacagc-3'	5'-tctgcctttcggtgaatacc-3'
4	b3974	coaA	25%	2 PCR-bands	5'-aagtagcgcgcattctatgg-3'	5'-acgcggaatagacaaacagg-3'
5	b3997	hemE	37.50%	2 PCR-bands	5'-gccgtgagcgttactaccc-3'	5'-agagcggttcgaatttaccg-3'
6	b0928	aspC	100%	correct k.o.	5'-gacaacaaactgggcgtagg-3'	5'-ctggatttctggcaaagtgc-3'
7	b2687	luxS	100%	correct k.o.	5'-cccgatctgactttctctgc-3'	5'-ctatcggcacgtcgataacc-3'
8	b2927	epd	100%	correct k.o.	5'-gccggtatcacttcacaagc-3'	5'-cttctcgcctttgttgaagc-3'
9	b3993	thiE	100%	correct k.o.	5'-tacctgcgtaaggaggaagc-3'	5'-actgtgtcagtcgctgttgg-3'
10	b0026	ileS	25%	no PCR product	5'-gttgcaatggacctttacgg-3'	5'-gctaataccaatcgcaataccg-3'
