# Supplementary material for: Machine learning based analyses on metabolic networks supports high-throughput knockout screens
Source: BMC Syst Biol. 2008 Jul 24;2:67. doi: 10.1186/1752-0509-2-67 (PMC2526078; doi:10.1186/1752-0509-2-67)
Supplement: Additional File 2 — Supplementary Table S2. Results of all Reactions. [file 1752-0509-2-67-S2.pdf]

Supplementary Table S2 Results of all Reactions
Given are the essentiality predictions due to the experiment
in LB rich medium (KEIO), flux balance analyses for reactions (FBA)
and our machine learning approach (ML)

EC number	Reaction ID	Keio	FBA	ML	GPR association	Drugbank ID
	R_14GLUCANabcpp	no	no	no	(b4034 and b4033 and b4032 and b4035)	-
	R_14GLUCANtexi	no	no	no	b4036	-
3.1.4.16	R_23PDE2pp	no	no	no	b4213	-
3.1.4.16	R_23PDE4pp	no	no	no	b4213	-
3.1.4.16	R_23PDE7pp	no	no	no	b4213	-
3.1.4.16	R_23PDE9pp	no	no	no	b4213	-
	R_2AGPA120tipp	no	no	no	b2835	-
	R_2AGPA140tipp	no	no	no	b2835	-
	R_2AGPA141tipp	no	no	no	b2835	-
	R_2AGPA160tipp	no	no	no	b2835	-
	R_2AGPA161tipp	no	no	no	b2835	-
	R_2AGPA180tipp	no	no	no	b2835	-
	R_2AGPA181tipp	no	no	no	b2835	-
	R_2AGPE120tipp	no	no	no	b2835	-
	R_2AGPE140tipp	no	no	no	b2835	-
	R_2AGPE141tipp	no	no	no	b2835	-
	R_2AGPE160tipp	no	no	no	b2835	-
	R_2AGPE161tipp	no	no	no	b2835	-
	R_2AGPE180tipp	no	no	no	b2835	-
	R_2AGPE181tipp	no	no	no	b2835	-
2.3.1.40	R_2AGPEAT120	no	no	no	b2836	-
2.3.1.40	R_2AGPEAT140	no	no	no	b2836	-
2.3.1.40	R_2AGPEAT141	no	no	no	b2836	-
2.3.1.40	R_2AGPEAT160	no	no	no	b2836	-
2.3.1.40	R_2AGPEAT161	no	no	no	b2836	-
2.3.1.40	R_2AGPEAT180	no	no	no	b2836	-
2.3.1.40	R_2AGPEAT181	no	no	no	b2836	-
	R_2AGPG120tipp	no	no	no	b2835	-
	R_2AGPG140tipp	no	no	no	b2835	-
	R_2AGPG141tipp	no	no	no	b2835	-
	R_2AGPG160tipp	no	no	no	b2835	-
	R_2AGPG161tipp	no	no	no	b2835	-
	R_2AGPG180tipp	no	no	no	b2835	-
	R_2AGPG181tipp	no	no	no	b2835	-
2.3.1.40	R_2AGPGAT120	no	no	no	b2836	-
2.3.1.40	R_2AGPGAT140	no	no	no	b2836	-
2.3.1.40	R_2AGPGAT141	no	no	no	b2836	-
2.3.1.40	R_2AGPGAT160	no	no	no	b2836	-
2.3.1.40	R_2AGPGAT161	no	no	no	b2836	-
2.3.1.40	R_2AGPGAT180	no	no	no	b2836	-
2.3.1.40	R_2AGPGAT181	no	no	no	b2836	-
	R_2DGLCNRx	no	no	no	b3553	-
1.1.1.215	R_2DGLCNRy	no	no	no	b3553	-
	R_2DGULRx	no	no	no	b3553	-
	R_2DGULRy	no	no	no	b3553	-
	R_3HCINNMH	no	no	no	b0347	-
	R_3HPPPNH	no	no	no	b0347	-
	R_3KGK	no	no	no	b3580	-
	R_3NTD2pp	no	no	no	b4213	-
	R_3NTD4pp	no	no	no	b4213	-
	R_3NTD7pp	no	no	no	b4213	-
	R_3NTD9pp	no	no	no	b4213	-
1.1.1.100	R_3OAR100	yes	yes	yes	b1093	DB03461,
1.1.1.100	R_3OAR120	yes	yes	yes	b1093	DB03461,
1.1.1.100	R_3OAR121	yes	yes	yes	b1093	DB03461,

1.1.1.100	R_3OAR140	yes	yes	yes	b1093	DB03461,
1.1.1.100	R_3OAR141	yes	yes	yes	b1093	DB03461,
1.1.1.100	R_3OAR160	yes	yes	yes	b1093	DB03461,
1.1.1.100	R_3OAR161	yes	yes	yes	b1093	DB03461,
1.1.1.100	R_3OAR180	yes	no	no	b1093	DB03461,
1.1.1.100	R_3OAR181	yes	no	yes	b1093	DB03461,
1.1.1.100	R_3OAR40	yes	yes	no	b1093	DB03461,
1.1.1.100	R_3OAR60	yes	yes	yes	b1093	DB03461,
1.1.1.100	R_3OAR80	yes	yes	yes	b1093	DB03461,
2.3.1.41	R_3OAS121	yes	yes	yes	b2323	DB02039,DB02316,DB03264,DB03600,DB03661,DB04302,DB04519,
2.3.1.41	R_3OAS141	yes	yes	yes	b2323	DB02039,DB02316,DB03264,DB03600,DB03661,DB04302,DB04519,
2.3.1.41	R_3OAS161	yes	yes	yes	b2323	DB02039,DB02316,DB03264,DB03600,DB03661,DB04302,DB04519,
2.3.1.41	R_3OAS181	no	no	no	b1095	DB02039,DB02316,DB03264,DB03600,DB03661,DB04302,DB04519,
	R_3PEPTabcpp	no	no	no	(b1329 and b1244 and b1245 and b1246 and b1247)	-
1.4.3.6	R_42A12BOOXpp	no	no	no	b1386	DB01634,DB01657,DB02511,DB02537,DB02928,DB03631,DB04334,
	R_4HTHRS	no	no	no	b0004	-
	R_4PCP	no	no	no	b1192	-
	R_4PEPTabcpp	no	no	no	(b1243 and b1244 and b1245 and b1246 and b1247)	-
1.1.1.69	R_5DGLCNR	no	no	no	b4266	-
	R_5DGLCNt2rpp	no	no	no	b4265	-
	R_5DOAN	no	no	no	b0159	-
	R_AADDGT	no	no	no	b4481	-
	R_AAMYL	no	no	no	b1927	-
	R_AAMYLpp	no	no	no	b3571	-
3.2.1.86	R_AB6PGH	no	no	no	b2901	-
1.2.1.19	R_ABUTD	no	no	no	b1444	-
	R_ABUTt2pp	no	no	no	b2663	-
	R_ACACCT	no	no	no	(b2221 and b2222)	-
2.3.1.9	R_ACACT1r	no	no	no	b2224	DB01915,DB02039,DB02160,DB03045,
	R_ACACt2pp	no	no	no	b2223	-
	R_ACALDtpp	no	no	no	s0001	-
2.3.1.118	R_ACANTHAT	no	no	no	b1463	-
	R_ACBIPGT	no	no	no	b1993	-
6.4.1.2	R_ACCOAC	yes	yes	no	(b0185 and b2316 and b3255 and b3256)	DB03781,
6.2.1.13	R_ACCOAL	no	no	no	b0335	-
2.7.1.59	R_ACGAMK	no	no	no	b1119	-
	R_ACGAMT	no	no	no	b3784	-
	R_ACGAptspp	no	no	no	((b2417 and b1101 and b2415 and b2416) or (b0679 and b2415 and b2416))	-
2.7.2.8	R_ACGK	no	yes	no	b3959	DB04184,DB04444,
2.3.1.1	R_ACGS	no	yes	yes	b2818	-
	R_ACMAMUT	no	no	no	b3794	-

	R_ACMANAptspp	no	no	no	(b1817 and b1818 and b1819 and b2415 and b2416)	-
	R_ACMUMptspp	no	no	no	(b2417 and b2429 and b2415 and b2416)	-
	R_ACNAMt2pp	no	no	no	b3224	-
4.1.3.3	R_ACNML	no	no	no	b3225	-
1.3.99.2	R_ACOAD1f	no	no	no	b0221	-
1.3.99.3	R_ACOAD2f	no	no	no	b0221	DB03415,
1.3.99.3	R_ACOAD3f	no	no	no	b0221	DB03415,
1.3.99.3	R_ACOAD4f	no	no	no	b0221	DB03415,
1.3.99.3	R_ACOAD5f	no	no	no	b0221	DB03415,
1.3.99.3	R_ACOAD6f	no	no	no	b0221	DB03415,
1.3.99.3	R_ACOAD7f	no	no	no	b0221	DB03415,
1.3.99.3	R_ACOAD8f	no	no	no	b0221	DB03415,
3.5.1.16	R_ACODA	no	yes	yes	b3957	-
5.3.3.7	R_ACONIs	no	no	no	s0001	-
	R_ACONMT	no	no	no	b1519	-
6.2.1.1	R_ACS	no	no	no	b4069	-
	R_ACt4pp	no	no	no	b4067	-
3.5.4.4	R_ADA	no	no	no	b1623	DB02096,DB02472,DB02616,DB02830,DB03015,DB03220,DB03370,DB03572,DB04218,
	R_ADCL	no	yes	no	b1096	-
	R_ADCS	no	yes	no	(b3360 and b1812)	-
3.5.4.2	R_ADD	no	no	no	b3665	-
	R_ADEt2rpp	no	no	no	b3654	-
2.7.4.3	R_ADK1	yes	no	no	b0474	-
	R_ADK3	yes	no	no	b0474	-
	R_ADK4	yes	no	no	b0474	-
4.1.1.50	R_ADMDC	no	no	no	b0120	DB03754,
4.6.1.1	R_ADNCYC	no	no	no	b3806	DB02355,DB02587,DB02596,DB04447,
2.7.1.20	R_ADNK1	yes	no	no	b0474	DB02416,
	R_ADNt2rpp	no	no	no	b2406	-
	R_ADNtex	no	no	no	b0411	-
3.2.2.8	R_ADNUC	no	no	no	b0030	-
	R_ADOCBIK	no	no	no	b1993	-
	R_ADOCBLabcpp	no	no	no	(b1711 and b1709 and b0158)	-
	R_ADOCBLS	no	no	no	b1992	-
	R_ADOCBLtonex	no	no	no	(b3966 and (b1252 and b3005 and b3006))	-
3.6.1.13	R_ADPRDP	no	no	no	b3397	DB01975,DB02059,DB04352,
2.4.2.7	R_ADPT	no	no	no	b0469	-
2.7.1.25	R_ADSK	no	yes	no	b2750	DB02661,DB04077,
4.3.2.2	R_ADSL1r	yes	yes	yes	b1131	-
4.3.2.2	R_ADSL2r	yes	yes	yes	b1131	-
6.3.4.4	R_ADSS	no	yes	yes	b4177	DB02109,DB02150,DB02493,DB02666,DB02682,DB02836,DB02954,DB03146,DB03510,DB04184,DB04460,
3.5.1.25	R_AGDC	no	no	no	b0677	-
	R_AGM3PA	no	no	no	b0110	-
	R_AGM3PH	no	no	no	b1107	-
	R_AGM3Pt2pp	no	no	no	b0433	-
	R_AGM4PA	no	no	no	b0110	-
	R_AGM4PCP	no	no	no	b1192	-
	R_AGM4PH	no	no	no	b1107	-
	R_AGM4Pt2pp	no	no	no	b0433	-

	R_AGMH	no	no	no	b1107	-
5.1.3.20	R_AGMHE	no	no	no	b3619	DB01774,DB03398,DB03461,
3.5.3.11	R_AGMT	no	no	no	b2937	-
	R_AGMt2pp	no	no	no	b0433	-
2.3.1.51	R_AGPAT120	yes	no	yes	b3018	-
2.3.1.51	R_AGPAT140	yes	no	yes	b3018	-
2.3.1.51	R_AGPAT141	yes	no	yes	b3018	-
2.3.1.51	R_AGPAT160	yes	yes	yes	b3018	-
2.3.1.51	R_AGPAT161	yes	yes	yes	b3018	-
2.3.1.51	R_AGPAT180	yes	no	yes	b3018	-
2.3.1.51	R_AGPAT181	yes	no	yes	b3018	-
1.2.1.38	R_AGPR	no	yes	yes	b3958	-
	R_AGt3	no	no	no	(b0572 and b0573 and b0574 and b0575)	-
3.2.2.9	R_AHCYSNS	no	yes	no	b0159	DB02158,DB02281,DB02933,
2.1.2.3	R_AICART	no	yes	yes	b4006	DB03442,DB04057,
	R_AIRC2	no	yes	no	b0522	-
	R_AIRC3	no	yes	yes	b0523	-
	R_AKGDH	no	no	no	(b0116 and b0726 and b0727)	-
	R_AKGt2rpp	no	no	no	b2587	-
	R_ALAabcpp	no	no	no	(b3454 and b3455 and b3457 and b3460 and b3456)	-
3.4.17.14	R_ALAALAD	no	no	no	b1488	-
	R_ALAGLUE	no	no	no	b1325	-
	R_ALAt2pp	no	no	no	b4208	-
	R_ALAt4pp	no	no	no	b0007	-
6.1.1.7	R_ALATRS	no	no	yes	b2697	-
1.1.1.1	R_ALCD19	no	no	no	b0356	DB01711,DB02131,DB02249,DB02721,DB02732,DB02822,DB02871,DB03020,DB03061,DB03168,DB03226,DB03527,DB03704,DB04065,DB04071,DB04105,DB04113,DB04184,DB04312,DB04399,DB04421,DB04447,DB04448,
1.2.1.39	R_ALDD19x	no	no	no	b1385	-
1.2.1.3	R_ALDD2x	no	no	no	b1300	DB04447,
1.2.1.4	R_ALDD2y	no	no	no	b3588	DB03461,
	R_ALDD3y	no	no	no	b3588	-
	R_ALLabcpp	no	no	no	(b4087 and b4088 and b4086)	-
	R_ALLK	yes	no	no	b4084	-
	R_ALLPI	no	no	no	b4090	-
3.5.3.9	R_ALLTAMH	no	no	no	b0516	-
3.5.2.5	R_ALLTN	no	no	no	b0512	-
	R_ALLTNt2rpp	no	no	no	b0511	-
	R_ALLULPE	no	no	no	b4085	-
	R_ALR4x	no	no	no	b3945	-
4.2.1.7	R_ALTRH	no	no	no	b3091	-
	R_AM3PA	no	no	no	b0110	-
	R_AM4PA	no	no	no	b0110	-
	R_AM4PCP	no	no	no	b1192	-
2.4.1.25	R_AMALT1	no	no	no	b3416	DB04439,
2.4.1.25	R_AMALT2	no	no	no	b3416	DB04439,
2.4.1.25	R_AMALT3	no	no	no	b3416	DB04439,
2.4.1.25	R_AMALT4	no	no	no	b3416	DB04439,
	R_AMANAPEr	no	no	no	b3223	-
2.7.1.60	R_AMANK	no	no	no	b3222	-
2.6.1.62	R_AMAOTr	no	no	no	b0774	DB02274,DB02725,DB04083,DB04402,
	R_AMMQLT8	no	no	no	b3833	-

	R_AMPMS2	no	yes	no	b3994	-
3.2.2.4	R_AMPN	no	no	no	b1982	DB03464,
	R_ANHMK	no	no	no	b1640	-
2.4.2.18	R_ANPRT	no	yes	no	b1263	DB02212,DB04184,
4.1.3.27	R_ANS	no	yes	no	(b1263 and b1264)	-
	R_AOBUTDs	no	no	no	s0001	-
2.3.1.47	R_AOXSr	no	no	no	b0776	DB03160,
3.6.1.41	R_AP4AH	no	no	no	b0049	-
	R_AP5AH	no	no	no	b0049	-
1.1.1.193	R_APRAUR	yes	yes	no	b0414	-
5.3.1.4	R_ARAI	no	no	no	b0062	-
	R_ARBabcpp	no	no	no	(b1901 and b1900 and b4460)	-
	R_ARBt2rpp	no	no	no	b2841	-
	R_ARBt3ipp	no	no	no	b1528	-
	R_ARBTNabcpp	no	no	no	(b0153 and b0151 and b0152)	-
	R_ARBTNexs	no	no	no	s0001	-
	R_ARGAGMt7pp	no	no	no	b4115	-
4.1.1.19	R_ARGDC	no	no	no	b4117	-
4.1.1.19	R_ARGDCpp	no	no	no	b2938	-
	R_ARGORNt7pp	no	no	no	b1605	-
4.3.2.1	R_ARGSL	no	yes	no	b3960	-
6.3.4.5	R_ARGSS	no	yes	yes	b3172	DB04077,
	R_ARGt3pp	no	no	no	b2923	-
6.1.1.19	R_ARGTRS	yes	no	yes	b1876	-
1.2.1.11	R_ASAD	yes	yes	no	b3433	DB03461,DB03502,DB04399,
	R_ASCBPL	no	no	no	b4192	-
	R_ASCBptspp	no	no	no	(b2415 and b2416 and b4195 and b4194 and b4193)	-
3.5.1.1	R_ASNNpp	no	no	no	b2957	DB01817,DB02233,DB03412,
6.3.5.4	R_ASNS1	no	no	no	b0674	-
6.3.1.1	R_ASNS2	no	no	no	b3744	-
	R_ASNt2rpp	no	no	no	b1453	-
6.1.1.22	R_ASNTRS	yes	no	yes	b0930	-
	R_ASO3t8pp	no	no	no	b3502	-
4.1.1.11	R_ASP1DC	no	yes	yes	b0131	DB03382,
	R_ASPabcpp	no	no	no	(b0655 and b0654 and b0653 and b0652)	-
2.1.3.2	R_ASPCT	no	yes	no	(b4244 and b4245)	DB03459,
	R_ASPO3	no	no	no	b2574	-
	R_ASPO4	no	no	no	b2574	-
	R_ASPO5	no	no	no	b2574	-
	R_ASPO6	no	no	no	b2574	-
4.3.1.1	R_ASPT	no	no	no	b4139	DB04184,
	R_ASPt2_2pp	no	no	no	b3528	-
	R_ASPt2pp	no	no	no	b4077	-
2.6.1.1	R_ASPTA	no	yes	no	b0928	DB01639,DB02142,DB02758,DB02783,DB02926,DB03629,DB03750,DB04083,DB04467,DB04762,DB04765,
6.1.1.12	R_ASPTRS	yes	no	yes	b1866	-
	R_ASR	no	no	no	(b3503 and b1064)	-
2.3.1.109	R_AST	no	no	no	b1747	-
	R_ATHRDHr	no	no	no	b1539	-
	R_ATPHs	no	no	no	s0001	-
2.4.2.17	R_ATPPRT	no	yes	no	b2019	-

3.6.3.14	R_ATPS4rpp	no	no	no	(((b3736 and b3737 and b3738) and (b3731 and b3732 and b3733 and b3734 and b3735)) or ((b3736 and b3737 and b3738) and (b3731 and b3732 and b3733 and b3734 and b3735) and b3739))	DB01218,DB02043,DB02686,DB03091,
	R_BALAt2pp	no	no	no	b4208	-
1.2.1.8	R_BETALDHx	no	no	no	b0312	-
1.2.1.8	R_BETALDHy	no	no	no	b0312	-
	R_BSORx	no	no	no	b3551	-
	R_BSORy	no	no	no	b3551	-
2.8.1.6	R_BTS4	no	no	no	b0775	DB03754,DB03775,
2.8.3.8	R_BUTCT	no	no	no	(b2221 and b2222)	-
	R_BUTt2rpp	no	no	no	b2223	-
	R_CA2t3pp	no	no	no	b1216	-
	R_CADVtpp	no	no	no	b4132	-
	R_CAt6pp	no	yes	yes	b3196	-
2.5.1.17	R_CBIAT	no	no	no	b1270	-
	R_CBItonex	no	no	no	(b3966 and (b1252 and b3005 and b3006))	-
	R_CBIuabcpp	no	no	no	(b1711 and b1709 and b0158)	-
	R_CBL1abcpp	no	no	no	(b1711 and b1709 and b0158)	-
	R_CBL1tonex	no	no	no	(b3966 and (b1252 and b3005 and b3006))	-
2.5.1.17	R_CBLAT	no	no	no	b1270	-
6.3.5.5	R_CBPS	no	no	no	(b0032 and b0033)	-
	R_CD2abcpp	no	no	no	b3469	-
	R_CD2tpp	no	no	no	b3040	-
3.6.1.26	R_CDAPPA120	no	no	no	b3918	DB04568,
3.6.1.26	R_CDAPPA140	no	no	no	b3918	DB04568,
3.6.1.26	R_CDAPPA141	no	no	no	b3918	DB04568,
3.6.1.26	R_CDAPPA160	no	no	no	b3918	DB04568,
3.6.1.26	R_CDAPPA161	no	no	no	b3918	DB04568,
3.6.1.26	R_CDAPPA180	no	no	no	b3918	DB04568,
3.6.1.26	R_CDAPPA181	no	no	no	b3918	DB04568,
	R_CDPMEK	yes	yes	yes	b1208	-
2.1.1.79	R_CFAS160E	no	no	no	b1661	DB00339,DB01718,DB04221,
2.1.1.79	R_CFAS160G	no	no	no	b1661	DB00339,DB01718,DB04221,
2.1.1.79	R_CFAS180E	no	no	no	b1661	DB00339,DB01718,DB04221,
2.1.1.79	R_CFAS180G	no	no	no	b1661	DB00339,DB01718,DB04221,
	R_CGLYabcpp	no	no	no	(b3544 and b3543 and b3542 and b3541 and b3540)	-
	R_CHLabcpp	no	no	no	(b2128 and b2129 and b2130 and b2131)	-
4.2.3.5	R_CHORS	no	yes	yes	b2329	DB03350,
	R_CHRPL	no	yes	no	b4039	-
	R_CINNDO	no	no	no	(b2538 and b2539 and b2540 and b2542)	-

4.1.3.6	R_CITL	no	no	no	((b0615 and b0616 and b0617) and b0614)	-
	R_CITt7pp	no	no	no	b0612	-
	R_CO2tpp	no	no	yes	s0001	-
	R_COBALT2abcpp	no	no	no	b3469	-
	R_COLIPAabcpp	yes	no	yes	b0914	-
	R_CPGNabcpp	no	no	no	(b0153 and b0151 and b0152)	-
	R_CPGNexs	no	no	no	s0001	-
	R_CPGNtonex	no	no	no	(b1102 and (b1252 and b3005 and b3006))	-
1.3.3.3	R_CPPPGO	no	no	no	b2436	-
	R_CPPPGO2	no	no	no	b3867	-
	R_CRNabcpp	no	no	no	(b2677 and b2678 and b2679)	-
	R_CRNBTCT	no	no	no	b0038	-
	R_CRNCAL2	no	no	no	b0037	-
	R_CRNCAR	no	no	no	b0036	-
	R_CRNCBCT	no	no	no	b0038	-
	R_CRNCDH	no	no	no	b0036	-
	R_CRNDabcpp	no	no	no	(b2677 and b2678 and b2679)	-
	R_CRNDCAL2	no	no	no	b0037	-
	R_CRNDt2rpp	no	no	no	b4111	-
	R_CRNt2rpp	no	no	no	b4111	-
	R_CRNt7pp	no	no	no	b0040	-
	R_CRNt8pp	no	no	no	b0040	-
	R_CS	no	yes	no	b0720	-
3.5.4.1	R_CSND	no	no	no	b0337	DB03939,DB04135,
	R_CSNt2pp	no	no	no	b0336	-
	R_CTBTabcpp	no	no	no	(b2677 and b2678 and b2679)	-
	R_CTBTCAL2	no	no	no	b0037	-
	R_CTBTt2rpp	no	no	no	b4111	-
6.3.4.2	R_CTPS2	yes	yes	no	b2780	-
	R_CU1abcpp	no	no	no	b0484	-
	R_CU1Opp	no	no	no	b0123	-
	R_CU2abcpp	no	no	no	b3469	-
	R_CU2tpp	no	yes	no	b3040	-
	R_CUt3	no	no	no	(b0572 and b0573 and b0574 and b0575)	-
2.8.1.1	R_CYANST	no	no	no	b3425	DB04053,DB04184,
2.8.1.1	R_CYANSTpp	no	no	no	b1308	DB04053,DB04184,
	R_CYNTAH	no	no	no	b0340	-
	R_CYNTt2pp	no	no	no	b0341	-
4.4.1.15	R_CYSDDS	no	no	no	b1919	-
4.1.1.12	R_CYSSADS	no	no	no	b2810	-
6.1.1.16	R_CYSTRS	yes	no	yes	b0526	-
	R_CYTBD2pp	no	no	no	(b0978 and b0979)	-
	R_CYTBO3_4pp	no	no	no	(b0429 and b0430 and b0431 and b0432)	-
3.5.4.5	R_CYTD	no	no	no	b2143	DB03068,DB03185,DB03562,DB04385,
	R_CYTDK2	no	no	no	b2066	-
	R_CYTDt2rpp	no	no	no	b2406	-
2.7.4.14	R_CYTK1	no	no	no	b0910	DB02456,DB02883,DB03664,DB04444,

2.7.4.14	R_CYTK2	no	no	yes	b0910	DB02456,DB02883,DB03664,DB04444,
1.4.99.1	R_DAAD	no	no	no	b1189	-
	R_DADA	no	no	no	b1623	-
2.7.4.11	R_DADK	yes	no	no	b0474	-
	R_DADNtex	no	no	no	b0411	-
2.7.1.107	R_DAGK120	no	no	no	b4042	-
2.7.1.107	R_DAGK140	no	no	no	b4042	-
2.7.1.107	R_DAGK141	no	no	no	b4042	-
2.7.1.107	R_DAGK160	no	no	no	b4042	-
2.7.1.107	R_DAGK161	no	no	no	b4042	-
2.7.1.107	R_DAGK180	no	no	no	b4042	-
2.7.1.107	R_DAGK181	no	no	no	b4042	-
	R_DALAt2pp	no	no	no	b4208	-
4.3.1.15	R_DAPAL	no	no	no	b2871	-
4.1.1.20	R_DAPDC	no	yes	yes	b2838	DB00548,DB03814,DB04083,
5.1.1.7	R_DAPE	no	yes	no	b3809	-
2.7.7.41	R_DASYN120	yes	no	yes	b0175	-
2.7.7.41	R_DASYN140	yes	no	yes	b0175	-
2.7.7.41	R_DASYN141	yes	no	yes	b0175	-
2.7.7.41	R_DASYN160	yes	yes	yes	b0175	-
2.7.7.41	R_DASYN161	yes	yes	yes	b0175	-
2.7.7.41	R_DASYN180	yes	no	yes	b0175	-
2.7.7.41	R_DASYN181	yes	no	yes	b0175	-
	R_DATPHs	no	no	no	s0001	-
	R_DB4PS	yes	yes	yes	b3041	-
6.3.3.3	R_DBTS	no	no	no	b0778	DB01715,DB02927,DB02941,DB03624,DB03775,
3.5.4.13	R_DCTPD	no	no	no	b2065	-
3.5.4.14	R_DCYTD	no	no	no	b2143	-
	R_DCYTtex	no	no	no	b0411	-
	R_DDCAtexi	no	no	no	b2344	-
2.7.1.58	R_DDGALK	no	no	no	b3693	-
	R_DDGLCNt2rpp	no	no	no	b3909	-
2.7.1.45	R_DDGLK	no	no	no	b3526	-
4.1.2.21	R_DDPGALA	no	no	no	b4477	-
2.7.4.8	R_DGK1	yes	no	no	b3648	-
	R_DGSNt2pp	no	no	no	b2964	-
4.2.1.9	R_DHAD1	no	yes	no	b3771	-
	R_DHAD2	no	yes	no	b3771	-
	R_DHAPT	no	no	no	(b1200 and b1199 and b1198 and b2415 and b2416)	-
1.3.1.28	R_DHBD	no	no	no	b0596	-
2.7.7.58	R_DHBS	no	no	no	b0594	-
	R_DHCIND	no	no	no	b2541	-
	R_DHCINDO	no	no	no	b0348	-
1.3.1.26	R_DHDPRy	yes	yes	yes	b0031	DB02575,DB03969,DB04184,
4.2.1.52	R_DHDPS	yes	yes	yes	b2478	-
6.3.2.12	R_DHFS	yes	yes	no	b2315	-
	R_DHNAOT4	no	no	no	b3930	-
	R_DHNPA2	no	yes	no	b3058	-
1.3.3.1	R_DHORD2	no	no	no	b0945	DB00263,DB00359,DB00664,DB01117,DB01145,DB02613,DB03122,DB03480,DB03523,DB03805,DB04184,DB04281,DB04583,
1.3.3.1	R_DHORD5	no	no	no	b0945	DB00263,DB00359,DB00664,DB01117,DB01145,DB02613,DB03122,DB03480,DB03523,DB03805,DB04184,DB04281,DB04583,
3.5.2.3	R_DHORTS	no	yes	yes	b1062	-
	R_DHPPD	no	no	no	b2541	-
3.5.4.26	R_DHPPDA2	yes	yes	yes	b0414	-

2.5.1.15	R_DHPS2	no	yes	yes	b3177	DB00250,DB00263,DB00576,DB00664,DB01298,DB03592,DB03705,DB04047,DB04196,
	R_DHPTDCs	no	yes	no	s0001	-
	R_DHPTPE	no	no	no	b2303	-
	R_DHQS	no	yes	no	b3389	-
4.2.1.10	R_DHQTi	no	yes	yes	b1693	DB02592,DB02786,DB02801,DB03739,DB03746,DB03868,DB04656,DB04698,
	R_DINSt2pp	no	no	no	b2964	-
	R_DKGLCNR2x	no	no	no	b3553	-
	R_DKGLCNR2y	no	no	no	b3553	-
2.5.1.1	R_DMATT	yes	yes	no	b0421	-
	R_DMPPS	yes	no	yes	b0029	-
	R_DMQMT	no	no	no	b2232	-
	R_DMSOR1pp	no	no	no	(b1872 and b1873)	-
	R_DMSOR2	no	no	no	(b0894 and b0895 and b0896)	-
	R_DMSOR2pp	no	no	no	(b1872 and b1873)	-
	R_DOGULNR	no	no	no	b3575	-
2.7.1.24	R_DPCOAK	no	yes	no	b0103	DB04184,
4.1.2.4	R_DRPA	no	no	no	b4381	DB04087,
	R_DSBAO1	no	no	no	(b3860 and b1185)	-
	R_DSBAO2	no	no	no	(b3860 and b1185)	-
	R_DSBCGT	no	no	no	b2893	-
	R_DSBDR	no	no	no	((b4136 and b3781) or (b4136 and b2582))	-
	R_DSBGGT	no	no	no	b0604	-
	R_DSERDHr	no	no	no	b1539	-
	R_DSERt2pp	no	no	no	b4208	-
2.7.4.9	R_DTMPK	yes	yes	yes	b1098	DB02452,DB03150,DB03195,DB03233,DB03280,DB03666,DB03845,DB03846,DB04184,
	R_DURIK1	no	no	no	b1238	-
	R_DURItex	no	no	no	b0411	-
	R_DXPRIi	yes	yes	yes	b0173	-
	R_DXPS	yes	yes	yes	b0420	-
	R_DXYLK	no	no	no	b3564	-
	R_E4PD	no	yes	yes	b2927	-
1.3.1.9	R_EAR100x	yes	no	yes	b1288	DB00609,DB00951,DB01691,DB01865,DB02990,DB03030,DB03534,DB04030,DB04289,DB04393,
1.3.1.10	R_EAR100y	yes	no	yes	b1288	-
1.3.1.9	R_EAR120x	yes	no	yes	b1288	DB00609,DB00951,DB01691,DB01865,DB02990,DB03030,DB03534,DB04030,DB04289,DB04393,
1.3.1.10	R_EAR120y	yes	no	yes	b1288	-
1.3.1.9	R_EAR121x	yes	no	yes	b1288	DB00609,DB00951,DB01691,DB01865,DB02990,DB03030,DB03534,DB04030,DB04289,DB04393,
1.3.1.10	R_EAR121y	yes	no	yes	b1288	-
1.3.1.9	R_EAR140x	yes	no	yes	b1288	DB00609,DB00951,DB01691,DB01865,DB02990,DB03030,DB03534,DB04030,DB04289,DB04393,

1.3.1.10	R_EAR140y	yes	no	yes	b1288	-
1.3.1.9	R_EAR141x	yes	no	yes	b1288	DB00609,DB00951,DB01691,DB01865,DB02990,DB03030,DB03534,DB04030,DB04289,DB04393,
1.3.1.10	R_EAR141y	yes	no	yes	b1288	-
1.3.1.9	R_EAR160x	yes	no	yes	b1288	DB00609,DB00951,DB01691,DB01865,DB02990,DB03030,DB03534,DB04030,DB04289,DB04393,
1.3.1.10	R_EAR160y	yes	no	yes	b1288	-
1.3.1.9	R_EAR161x	yes	no	yes	b1288	DB00609,DB00951,DB01691,DB01865,DB02990,DB03030,DB03534,DB04030,DB04289,DB04393,
1.3.1.10	R_EAR161y	yes	no	yes	b1288	-
1.3.1.9	R_EAR180x	yes	no	yes	b1288	DB00609,DB00951,DB01691,DB01865,DB02990,DB03030,DB03534,DB04030,DB04289,DB04393,
1.3.1.10	R_EAR180y	yes	no	yes	b1288	-
1.3.1.9	R_EAR181x	yes	no	yes	b1288	DB00609,DB00951,DB01691,DB01865,DB02990,DB03030,DB03534,DB04030,DB04289,DB04393,
1.3.1.10	R_EAR181y	yes	no	yes	b1288	-
1.3.1.9	R_EAR40x	yes	no	yes	b1288	DB00609,DB00951,DB01691,DB01865,DB02990,DB03030,DB03534,DB04030,DB04289,DB04393,
1.3.1.10	R_EAR40y	yes	no	yes	b1288	-
1.3.1.9	R_EAR60x	yes	no	yes	b1288	DB00609,DB00951,DB01691,DB01865,DB02990,DB03030,DB03534,DB04030,DB04289,DB04393,
1.3.1.10	R_EAR60y	yes	no	yes	b1288	-
1.3.1.9	R_EAR80x	yes	no	yes	b1288	DB00609,DB00951,DB01691,DB01865,DB02990,DB03030,DB03534,DB04030,DB04289,DB04393,
1.3.1.10	R_EAR80y	yes	no	yes	b1288	-
	R_ECA4OALpp	no	no	no	b3622	-
	R_ECAtpp	no	no	no	b3792	-
4.1.2.14	R_EDA	no	no	no	b1850	DB04184,
4.2.1.12	R_EDD	no	no	no	b1851	-
	R_EDTXS1	no	no	no	b1054	-
	R_EDTXS2	no	no	no	b1855	-
	R_EDTXS3	no	no	no	b2378	-
	R_EDTXS4	no	no	no	b1855	-
4.2.1.11	R_ENO	yes	no	no	b2779	DB03645,DB04077,
	R_ENTCS	yes	no	no	b0583	-
	R_ENTERES	no	no	no	b0585	-
	R_ENTERES2	no	no	no	b0585	-
4.3.1.7	R_ETHAAL	no	no	no	(b2440 and b2441)	-
	R_ETHSO3abcpp	no	no	no	(b0936 and b0933 and b0934)	-
	R_F6PP	no	no	no	b0822	-
	R_F6Pt6_2pp	no	no	no	b3666	-
3.1.2.14	R_FA100ACPHi	no	no	no	b0404	-
3.1.2.14	R_FA120ACPHi	no	no	no	b0404	-
3.1.2.14	R_FA140ACPHi	no	no	no	b0404	-
3.1.2.14	R_FA141ACPHi	no	no	no	b0404	-

3.1.2.14	R_FA160ACPHi	no	no	no	b0404	-
3.1.2.14	R_FA161ACPHi	no	no	no	b0404	-
3.1.2.14	R_FA80ACPHi	no	no	no	b0404	-
	R_FACOAE100	no	no	no	b0452	-
	R_FACOAE120	no	no	no	b0452	-
	R_FACOAE140	no	no	no	b0452	-
	R_FACOAE141	no	no	no	b0452	-
	R_FACOAE160	no	no	no	b0452	-
	R_FACOAE161	no	no	no	b0452	-
	R_FACOAE180	no	no	no	b0452	-
	R_FACOAE181	no	no	no	b0452	-
	R_FACOAE60	no	no	no	b0452	-
	R_FACOAE80	no	no	no	b0452	-
	R_FADRx	no	no	no	b3844	-
	R_FADRx2	no	no	no	(b2763 and b2764)	-
	R_FALDH2	no	no	no	b0356	-
	R_FALDtpp	no	no	no	s0001	-
	R_FALGTHLs	no	no	no	s0001	-
5.3.1.25	R_FCI	no	no	no	b2802	DB03815,
2.7.1.51	R_FCLK	no	no	no	b2803	-
4.99.1.1	R_FCLT	yes	yes	yes	b0475	DB01911,DB02188,
	R_FDMO	no	no	no	b0935	-
	R_FDMO2	no	no	no	b0935	-
	R_FDMO3	no	no	no	b0935	-
	R_FDMO4	no	no	no	b0935	-
	R_FDMO6	no	no	no	b0935	-
	R_FE2abcpp	no	no	no	b3409	-
	R_FE2t2pp	no	no	no	b2392	-
	R_FE2t3pp	no	no	no	b3915	-
	R_FE2tpp	no	no	no	b3040	-
	R_FE3DCITabcpp	no	no	no	(b4290 and b4289 and b4288 and b4287)	-
	R_FE3DCITtonex	no	no	no	(b4291 and (b1252 and b3005 and b3006))	-
	R_FE3DHBZSabcpp	no	no	no	(b0592 and b0588 and b0590 and b0589)	-
	R_FE3DHBZStonex	no	no	no	((b0805 and (b1252 and b3005 and b3006)) or (b2155 and (b1252 and b3005 and b3006)))	-
	R_FE3HOXabcpp	no	no	no	(b0153 and b0151 and b0152)	-
	R_FE3HOXexs	no	no	no	s0001	-
	R_FE3HOXtonex	no	no	no	(b0150 and (b1252 and b3005 and b3006))	-
	R_FE3Ri	no	no	no	b3844	-
	R_FECRMabcpp	no	no	no	(b0153 and b0151 and b0152)	-
	R_FECRMexs	no	no	no	s0001	-
	R_FECRMtonex	no	no	no	(b0150 and (b1252 and b3005 and b3006))	-
	R_FEENTERabcpp	no	no	no	(b0592 and b0588 and b0590 and b0589)	-
	R_FEENTERexs	no	no	no	s0001	-

	R_FEENTERtonex	no	no	no	(b0584 and (b1252 and b3005 and b3006))	-
	R_FEENTERtpp	no	no	no	b0591	-
	R_FEOXAMabcpp	no	no	no	(b0153 and b0151 and b0152)	-
	R_FEOXAMexs	no	no	no	s0001	-
	R_FEOXAMtonex	no	no	no	(b0150 and (b1252 and b3005 and b3006))	-
1.16.3.1	R_FEROpp	no	no	no	b0123	-
	R_FHL	no	no	no	((b4079 and (b2481 and b2482 and b2483 and b2484 and b2485 and b2486 and b2487 and b2488 and b2489 and b2490)) or (b4079 and (b2719 and b2720 and b2721 and b2722 and b2723 and b2724)))	-
1.18.1.2	R_FLDR	no	no	no	((b0684 and b3924) or (b2895 and b3924))	DB01753,DB03461,DB04184,
1.5.1.30	R_FLVRx	no	no	no	b3844	DB03461,DB04363,
2.1.2.9	R_FMETTRS	yes	no	no	b3288	-
2.7.7.2	R_FMNAT	yes	yes	yes	b0025	-
1.3.99.1	R_FRD2	no	no	no	(b4151 and b4152 and b4153 and b4154)	DB00730,DB03014,DB03343,DB04631,
1.3.99.1	R_FRD3	no	no	no	(b4151 and b4152 and b4153 and b4154)	DB00730,DB03014,DB03343,DB04631,
2.7.1.56	R_FRUK	no	no	no	b2168	-
	R_FRULYSDG	no	no	no	b3371	-
	R_FRULYSE	no	no	no	b4474	-
	R_FRULYSK	no	no	no	b3374	-
	R_FRULYSt2pp	no	no	no	b3370	-
	R_FRUpts2pp	no	no	no	(b1817 and b1818 and b1819 and b2415 and b2416)	-
	R_FRUptspp	no	no	no	(b2167 and b2169 and b2415 and b2416)	-
	R_FRUURt2rpp	no	no	no	b4321	-
3.5.1.10	R_FTHFD	no	no	no	b1232	-
	R_FUCtpp	no	no	no	b2801	-
	R_FUMt2_2pp	no	no	no	b3528	-
2.3.1.157	R_G1PACT	yes	yes	no	b3730	-
3.1.3.10	R_G1PPpp	no	no	no	b1002	-
5.4.3.8	R_G1SAT	yes	yes	no	b0154	DB02054,DB02142,
	R_G2PPpp	no	no	no	b4055	-
2.3.1.15	R_G3PAT120	yes	no	yes	b4041	-
2.3.1.15	R_G3PAT140	yes	no	yes	b4041	-
2.3.1.15	R_G3PAT141	yes	no	yes	b4041	-
2.3.1.15	R_G3PAT160	yes	yes	yes	b4041	-
2.3.1.15	R_G3PAT161	yes	yes	yes	b4041	-
2.3.1.15	R_G3PAT180	yes	no	yes	b4041	-
2.3.1.15	R_G3PAT181	yes	no	yes	b4041	-

	R_G3PCabcpp	no	no	no	(b3452 and b3453 and b3450 and b3451)	-
1.1.1.94	R_G3PD2	yes	yes	no	b3608	-
1.1.99.5	R_G3PD6	no	no	no	(b2241 and b2242 and b2243)	-
1.1.99.5	R_G3PD7	no	no	no	(b2241 and b2242 and b2243)	-
	R_G3PEabcpp	no	no	no	(b3452 and b3453 and b3450 and b3451)	-
	R_G3PGabcpp	no	no	no	(b3452 and b3453 and b3450 and b3451)	-
	R_G3PIabcpp	no	no	no	(b3452 and b3453 and b3450 and b3451)	-
	R_G3PSabcpp	no	no	no	(b3452 and b3453 and b3450 and b3451)	-
	R_G3PT	no	no	no	b0822	-
	R_G5SADs	no	yes	yes	s0001	-
1.2.1.41	R_G5SD	no	no	no	b0243	-
3.5.99.6	R_G6PDA	no	no	no	b0678	DB02171,DB02445,DB04277,
1.1.1.49	R_G6PDH2r	no	no	no	b1852	DB03461,
3.1.3.9	R_G6PP	no	no	no	b0822	-
	R_G6Pt6_2pp	no	no	no	b3666	-
3.1.3.10	R_GAL1PPpp	no	no	no	b1002	-
	R_GALabcpp	no	no	no	(b2149 and b2150 and b2148)	-
4.2.1.42	R_GALCTD	no	no	no	b3128	-
	R_GALCTLO	no	no	no	b4358	-
4.2.1.6	R_GALCTND	no	no	no	b4478	-
	R_GALCTNLt2pp	no	no	no	b4356	-
	R_GALCTNt2pp	no	no	no	b3691	-
5.1.3.3	R_GALM2pp	no	no	no	b0756	DB01861,
3.2.1.22	R_GALS3	no	no	no	b4119	DB03965,
	R_GALT1	no	no	no	b3628	-
	R_GALt2pp	no	no	no	b2943	-
	R_GALTptspp	no	no	no	(b2094 and b2093 and b2092 and b2415 and b2416)	-
2.7.7.9	R_GALUi	no	no	no	b1236	-
	R_GALURt2rpp	no	no	no	b3093	-
	R_GAM6Pt6_2pp	no	no	no	b3666	-
	R_GAMptspp	no	no	no	(b1817 and b1818 and b1819 and b2415 and b2416)	-
1.2.1.12	R_GAPD	yes	no	no	b1779	DB02059,DB02205,DB03211,DB03331,DB03814,DB03893,DB04477,
2.1.2.2	R_GARFT	no	no	no	b2500	DB02540,DB02794,DB03546,DB04264,
	R_GART	no	no	no	b1849	-
1.2.1.21	R_GCALDD	no	yes	no	b1415	-
	R_GDMANE	no	no	no	b2052	-
	R_GDPMNH	no	no	no	b2051	-
	R_GDPMNP	no	no	no	b2467	-
2.6.1.16	R_GF6PTA	yes	yes	no	b3729	DB02445,DB02446,DB03814,DB04184,
	R_GGGABADr	no	no	no	b1300	-
	R_GGGABAH	no	no	no	b1298	-
	R_GGPTRCO	no	no	no	b1301	-

	R_GGPTRCS	no	no	no	b1297	-
2.1.2.1	R_GHMT2r	no	no	no	b2551	DB02067,DB02718,DB02800,DB02824,
2.7.4.8	R_GK1	yes	yes	yes	b3648	-
2.4.1.18	R_GLBRAN2	no	no	no	b3432	-
	R_GLCabcpp	no	no	no	(b2149 and b2150 and b2148)	-
2.3.1.79	R_GLCATr	no	no	no	b0459	-
	R_GLCptspp	no	no	no	((b2417 and b1101 and b2415 and b2416) or (b1817 and b1818 and b1819 and b2415 and b2416) or (b2417 and b1621 and b2415 and b2416))	-
4.1.2.20	R_GLCRAL	no	no	no	b3126	-
2.4.1.21	R_GLCS1	no	no	no	b3429	-
	R_GLCt2pp	no	no	no	b2943	-
	R_GLCtexi	no	no	no	b4036	-
	R_GLCTR1	no	no	no	b3631	-
	R_GLCTR2	no	no	no	b3627	-
	R_GLCTR3	no	no	no	b3626	-
	R_GLDBRAN2	no	no	no	b3431	-
2.7.7.27	R_GLGC	no	no	no	b3430	DB01774,DB02983,
	R_GLNabcpp	no	no	no	(b0811 and b0810 and b0809)	-
6.1.1.18	R_GLNTRS	yes	no	yes	b0680	-
	R_GLTPD	no	no	no	b2091	-
2.7.2.11	R_GLU5K	no	no	no	b0242	-
	R_GLUabcpp	no	no	no	(b0655 and b0654 and b0653 and b0652)	-
	R_GLUABUTt7pp	no	no	no	b1492	-
6.3.2.2	R_GLUCYS	no	no	no	b2688	-
1.4.1.4	R_GLUDy	no	no	no	b1761	-
3.5.1.2	R_GLUNpp	no	no	no	b2957	DB04522,
2.4.2.14	R_GLUPRT	no	yes	no	b2312	DB03942,DB04296,
5.1.1.3	R_GLUR	yes	yes	no	b3967	-
1.4.1.13	R_GLUSy	no	no	no	(b3212 and b3213)	-
	R_GLUt2rpp	no	no	no	b4077	-
	R_GLUt4pp	no	no	no	b3653	-
	R_GLUTRR	yes	yes	yes	b1210	-
6.1.1.17	R_GLUTRS	yes	yes	yes	b2400	-
4.1.1.47	R_GLXCL	no	no	no	b0507	-
	R_GLYALDtpp	no	no	no	b3927	-
2.3.1.29	R_GLYAT	no	no	no	b3617	-
	R_GLYBabcpp	no	no	no	(b2128 and b2129 and b2130 and b2131)	-
	R_GLYBt2pp	no	no	no	b1801	-
	R_GLYC3Pabcpp	no	no	no	(b3452 and b3453 and b3450 and b3451)	-
	R_GLYC3Pt6pp	no	no	no	b2240	-
1.1.1.6	R_GLYCDx	no	no	no	b3945	-
2.7.1.31	R_GLYCK	no	no	no	b0514	-
2.7.1.31	R_GLYCK2	no	no	no	b3124	-
	R_GLYCL	no	no	no	(b2904 and b2903 and b2905 and b0116)	-

	R_GLYCLTt4pp	no	no	no	b4067	-
	R_GLYCTO2	no	no	no	(b2979 and b4467 and b4468)	-
	R_GLYCTO3	no	no	no	(b2979 and b4467 and b4468)	-
	R_GLYCTO4	no	no	no	(b2979 and b4467 and b4468)	-
2.7.1.30	R_GLYK	no	no	no	b3926	DB02937,
3.1.2.6	R_GLYOX	no	no	no	b0212	DB03889,DB04184,DB04399,
	R_GLYt4pp	no	no	no	b0007	-
4.2.1.47	R_GMAND	no	no	no	b2053	-
	R_GMHEPAT	no	no	no	b3052	-
	R_GMHEPK	no	no	no	b3052	-
	R_GMHEPPA	no	no	no	b0200	-
1.7.1.7	R_GMPR	no	no	no	b0104	-
6.3.5.2	R_GMPS2	no	yes	no	b2507	DB02212,
1.1.1.44	R_GND	no	no	no	b2029	DB02212,DB03962,
	R_GOFUCR	no	no	no	b2052	-
	R_GP4GH	no	no	no	b0049	-
3.1.4.46	R_GPDDA1	no	no	no	b3449	-
3.1.4.46	R_GPDDA1pp	no	no	no	b2239	-
3.1.4.46	R_GPDDA2	no	no	no	b3449	-
3.1.4.46	R_GPDDA2pp	no	no	no	b2239	-
3.1.4.46	R_GPDDA3	no	no	no	b3449	-
3.1.4.46	R_GPDDA3pp	no	no	no	b2239	-
3.1.4.46	R_GPDDA4	no	no	no	b3449	-
3.1.4.46	R_GPDDA4pp	no	no	no	b2239	-
3.1.4.46	R_GPDDA5	no	no	no	b3449	-
3.1.4.46	R_GPDDA5pp	no	no	no	b2239	-
2.5.1.10	R_GRTT	yes	yes	yes	b0421	DB02782,
	R_GSNK	no	no	no	b0477	-
	R_GSNt2pp	no	no	no	b2964	-
3.5.1.78	R_GSPMDA	no	no	no	b2988	-
6.3.1.8	R_GSPMDS	no	no	no	b2988	-
1.8.1.7	R_GTHOr	no	no	no	b3500	DB00336,DB01644,DB02553,DB02895,DB03310,DB03867,
	R_GTHRDabcpp	no	no	no	(b0829 and b0830 and b0831 and b0832)	-
	R_GTHRDHpp	no	no	no	b3447	-
6.3.2.3	R_GTHS	no	no	no	b2947	-
3.5.4.16	R_GTPCI	yes	yes	yes	b2153	-
	R_GTPCII2	yes	yes	yes	b1277	-
3.6.1.40	R_GTPDPDP	no	no	no	b3779	-
2.7.6.5	R_GTPDPK	no	no	no	b2784	DB02836,
	R_GTPHs	no	no	no	s0001	-
3.5.4.3	R_GUAD	no	no	no	b2883	-
	R_GUAt2pp	no	no	no	b3654	-
	R_GUAtex	no	no	no	b0411	-
5.3.1.12	R_GUI1	no	no	no	b3092	-
5.3.1.12	R_GUI2	no	no	no	b3092	-
	R_H2St1pp	no	no	no	s0001	-
	R_H2tpp	no	no	no	s0001	-
	R_HBZOPT	yes	yes	no	b4040	-
	R_HCINNMt2rpp	no	no	no	b0353	-
2.1.1.10	R_HCYSMT	no	no	no	b0261	-
	R_HCYSMT2	no	no	no	b0261	-
	R_HDCAtexi	no	no	no	b2344	-
	R_HDCEAtexi	no	no	no	b2344	-
5.3.3.8	R_HDCOAI	no	no	no	b3846	-
	R_HEMEOS	no	no	no	b0428	-
	R_HEPK1	no	no	no	b3630	-
	R_HEPK2	no	no	no	b3625	-

	R_HEPT1	no	no	no	b3621	-
	R_HEPT2	no	no	no	b3620	-
	R_HEPT3	no	no	no	b3632	-
	R_HEPT4	yes	no	no	b3623	-
2.7.1.50	R_HETZK	no	no	no	b2104	DB01769,
2.7.1.1	R_HEX1	no	no	no	b2388	-
2.7.1.1	R_HEX7	no	no	no	b0394	-
	R_HEXt2rpp	no	no	no	b2223	-
	R_HG2abcpp	no	no	no	b3469	-
	R_HG2t3pp	no	no	no	b3915	-
	R_HISabcpp	no	no	no	(b2309 and b2307 and b2306 and b2308)	-
	R_HISt2rpp	no	no	no	b0112	-
1.1.1.23	R_HISTD	no	yes	no	b2020	DB04077,DB04447,
3.1.3.15	R_HISTP	no	yes	no	b2022	-
6.1.1.21	R_HISTRS	yes	no	yes	b2514	-
	R_HKNDDH	no	no	no	b0349	-
	R_HKNTDH	no	no	no	b0349	-
4.3.1.8	R_HMBS	yes	yes	yes	b3805	-
	R_HOPNTAL	no	no	no	b0352	-
	R_HPPK2	yes	yes	no	b0142	-
1.13.11.16	R_HPPPNDO	no	no	no	b0348	-
	R_HPPPNt2rpp	no	no	no	b0353	-
5.3.1.22	R_HPYRI	no	no	no	b0508	-
2.7.1.39	R_HSK	no	yes	no	b0003	DB01660,
2.3.1.46	R_HSST	no	yes	no	b4013	-
2.6.1.9	R_HSTPT	no	yes	no	b2021	DB01813,DB02142,DB03997,
	R_HXAND	no	no	no	(b2866 and b2867 and b2868)	-
2.8.3.8	R_HXCT	no	no	no	(b2221 and b2222)	-
1.1.1.42	R_ICDHyr	no	yes	no	b1136	DB03461,DB03806,DB04530,
5.4.99.6	R_ICHORS	no	no	no	b2265	-
	R_ICHORSi	no	no	no	b0593	-
3.3.2.1	R_ICHORT	no	no	no	b0595	-
4.1.3.1	R_ICL	no	no	no	b4015	-
	R_IDOND	no	no	no	b4267	-
	R_IDOND2	no	no	no	b4267	-
	R_IDONt2rpp	no	no	no	b4265	-
	R_IG3PS	no	yes	yes	(b2023 and b2025)	-
4.2.1.19	R_IGPDH	no	yes	no	b2022	-
4.1.1.48	R_IGPS	no	yes	no	b1262	DB03543,DB04143,
	R_ILEabcpp	no	no	no	(b3454 and b3455 and b3457 and b3460 and b3456)	-
	R_ILEt2rpp	no	no	no	b0401	-
2.6.1.42	R_ILETA	no	yes	no	b3770	DB01813,DB02142,DB02635,DB03993,DB04063,
6.1.1.5	R_ILETRS	no	no	yes	b0026	DB00410,DB01755,
3.5.4.10	R_IMPC	no	yes	no	b4006	DB03442,DB04057,
1.1.1.205	R_IMPD	no	no	no	b2508	DB00811,DB01693,DB01915,DB01945,DB03055,DB03070,DB03122,DB03948,
	R_INDOLEt2pp	no	no	no	(b3265 and b3266)	-
	R_INDOLEt2rpp	no	no	no	b3161	-
	R_INOSTt4pp	no	no	no	b3679	-
3.2.2.8	R_INSH	no	no	no	b0030	-
2.7.1.73	R_INSK	no	no	no	b0477	-
	R_INSt2pp	no	no	no	b2964	-
	R_INSt2rpp	no	no	no	b2406	-

	R_INStex	no	no	no	b0411	-
5.3.3.2	R_IPDDI	no	no	no	b2889	DB01799,DB02480,DB03165,DB04170,
	R_IPDPS	yes	no	no	b0029	-
1.1.1.85	R_IPMD	no	yes	no	b0073	-
4.2.1.33	R_IPPMIa	no	yes	no	(b0071 and b0072)	-
4.2.1.33	R_IPPMIb	no	yes	no	(b0071 and b0072)	-
4.1.3.12	R_IPPS	no	yes	no	b0074	-
	R_K2L4Aabcpp	yes	yes	yes	b0914	-
	R_Kabcpp	no	no	no	(b0698 and b0697 and b0696)	-
1.1.1.86	R_KARA1	no	yes	no	b3774	DB03387,DB04497,
1.1.1.86	R_KARA2	no	yes	yes	b3774	DB03387,DB04497,
	R_KAS15	no	no	no	b1091	-
2.7.7.38	R_KDOCT2	yes	yes	yes	b0918	DB02344,DB04482,
3.1.3.45	R_KDOPP	no	yes	yes	b3198	DB03814,
4.1.2.16	R_KDOPS	yes	yes	yes	b1215	-
1.1.2.3	R_L_LACD2	no	no	no	b3605	DB02164,DB03014,
1.1.2.3	R_L_LACD3	no	no	no	b3605	DB02164,DB03014,
	R_LA4NTpp	no	no	no	b2257	-
3.2.1.23	R_LACZ	no	no	no	b0344	DB01862,DB01920,DB02228,DB02294,DB02376,DB02525,DB02632,DB02748,DB03398,DB04155,DB04184,DB04282,DB04382,DB04530,
3.2.1.108	R_LACZpp	no	no	no	b2132	-
	R_LADGMDH	no	no	no	b1326	-
1.1.1.21	R_LALDO2x	no	no	no	b3945	DB01689,DB02020,DB02021,DB02101,DB02132,DB02518,DB02712,DB02834,DB02994,DB03461,
	R_LALGP	no	no	no	b0237	-
1.2.1.21	R_LCADi	no	no	no	b1415	-
1.1.1.77	R_LCARS	no	no	no	b2799	DB02059,
	R_LCTStpp	no	no	no	b0343	-
1.1.2.4	R_LDH_D2	no	no	no	b2133	-
	R_LEUabcpp	no	no	no	((b3454 and b3455 and b3457 and b3460 and b3456) or (b3454 and b3455 and b3457 and b3458 and b3456))	-
	R_LEUt2rpp	no	no	no	b0401	-
6.1.1.4	R_LEUTRS	yes	no	yes	b0642	-
4.4.1.5	R_LGTHL	no	no	no	b1651	DB03130,DB03330,DB03602,DB04132,
	R_LIPAabcpp	yes	no	yes	b0914	-
	R_LIPACabcpp	yes	no	yes	b0914	-
	R_LIPAHT2ex	no	no	no	b0622	-
	R_LIPAHTex	no	no	no	b0622	-
2.4.1.182	R_LPADSS	yes	yes	no	b0182	-
3.1.1.5	R_LPLIPAL1A120pp	no	no	no	b0494	DB02364,DB02983,DB04519,
3.1.1.5	R_LPLIPAL1A140pp	no	no	no	b0494	DB02364,DB02983,DB04519,
3.1.1.5	R_LPLIPAL1A141pp	no	no	no	b0494	DB02364,DB02983,DB04519,
3.1.1.5	R_LPLIPAL1A160pp	no	no	no	b0494	DB02364,DB02983,DB04519,

3.1.1.5	R_LPLIPAL1A161pp	no	no	no	b0494	DB02364,DB02983,DB04519,
3.1.1.5	R_LPLIPAL1A180pp	no	no	no	b0494	DB02364,DB02983,DB04519,
3.1.1.5	R_LPLIPAL1A181pp	no	no	no	b0494	DB02364,DB02983,DB04519,
3.1.1.5	R_LPLIPAL1E120pp	no	no	no	b0494	DB02364,DB02983,DB04519,
3.1.1.5	R_LPLIPAL1E140pp	no	no	no	b0494	DB02364,DB02983,DB04519,
3.1.1.5	R_LPLIPAL1E141pp	no	no	no	b0494	DB02364,DB02983,DB04519,
3.1.1.5	R_LPLIPAL1E160pp	no	no	no	b0494	DB02364,DB02983,DB04519,
3.1.1.5	R_LPLIPAL1E161pp	no	no	no	b0494	DB02364,DB02983,DB04519,
3.1.1.5	R_LPLIPAL1E180pp	no	no	no	b0494	DB02364,DB02983,DB04519,
3.1.1.5	R_LPLIPAL1E181pp	no	no	no	b0494	DB02364,DB02983,DB04519,
3.1.1.5	R_LPLIPAL1G120pp	no	no	no	b0494	DB02364,DB02983,DB04519,
3.1.1.5	R_LPLIPAL1G140pp	no	no	no	b0494	DB02364,DB02983,DB04519,
3.1.1.5	R_LPLIPAL1G141pp	no	no	no	b0494	DB02364,DB02983,DB04519,
3.1.1.5	R_LPLIPAL1G160pp	no	no	no	b0494	DB02364,DB02983,DB04519,
3.1.1.5	R_LPLIPAL1G161pp	no	no	no	b0494	DB02364,DB02983,DB04519,
3.1.1.5	R_LPLIPAL1G180pp	no	no	no	b0494	DB02364,DB02983,DB04519,
3.1.1.5	R_LPLIPAL1G181pp	no	no	no	b0494	DB02364,DB02983,DB04519,
3.1.1.5	R_LPLIPAL2A120	no	no	no	b3825	DB02364,DB02983,DB04519,
3.1.1.5	R_LPLIPAL2A140	no	no	no	b3825	DB02364,DB02983,DB04519,
3.1.1.5	R_LPLIPAL2A141	no	no	no	b3825	DB02364,DB02983,DB04519,
3.1.1.5	R_LPLIPAL2A160	no	no	no	b3825	DB02364,DB02983,DB04519,
3.1.1.5	R_LPLIPAL2A161	no	no	no	b3825	DB02364,DB02983,DB04519,
3.1.1.5	R_LPLIPAL2A180	no	no	no	b3825	DB02364,DB02983,DB04519,
3.1.1.5	R_LPLIPAL2A181	no	no	no	b3825	DB02364,DB02983,DB04519,
	R_LPLIPAL2ATE120	no	no	no	b3825	-
	R_LPLIPAL2ATE140	no	no	no	b3825	-
	R_LPLIPAL2ATE141	no	no	no	b3825	-
	R_LPLIPAL2ATE160	no	no	no	b3825	-
	R_LPLIPAL2ATE161	no	no	no	b3825	-
	R_LPLIPAL2ATE180	no	no	no	b3825	-
	R_LPLIPAL2ATE181	no	no	no	b3825	-
	R_LPLIPAL2ATG120	no	no	no	b3825	-
	R_LPLIPAL2ATG140	no	no	no	b3825	-
	R_LPLIPAL2ATG141	no	no	no	b3825	-
	R_LPLIPAL2ATG160	no	no	no	b3825	-

	R_LPLIPAL2ATG161	no	no	no	b3825	-
	R_LPLIPAL2ATG180	no	no	no	b3825	-
	R_LPLIPAL2ATG181	no	no	no	b3825	-
3.1.1.5	R_LPLIPAL2E120	no	no	no	b3825	DB02364,DB02983,DB04519,
3.1.1.5	R_LPLIPAL2E140	no	no	no	b3825	DB02364,DB02983,DB04519,
3.1.1.5	R_LPLIPAL2E141	no	no	no	b3825	DB02364,DB02983,DB04519,
3.1.1.5	R_LPLIPAL2E160	no	no	no	b3825	DB02364,DB02983,DB04519,
3.1.1.5	R_LPLIPAL2E161	no	no	no	b3825	DB02364,DB02983,DB04519,
3.1.1.5	R_LPLIPAL2E180	no	no	no	b3825	DB02364,DB02983,DB04519,
3.1.1.5	R_LPLIPAL2E181	no	no	no	b3825	DB02364,DB02983,DB04519,
3.1.1.5	R_LPLIPAL2G120	no	no	no	b3825	DB02364,DB02983,DB04519,
3.1.1.5	R_LPLIPAL2G140	no	no	no	b3825	DB02364,DB02983,DB04519,
3.1.1.5	R_LPLIPAL2G141	no	no	no	b3825	DB02364,DB02983,DB04519,
3.1.1.5	R_LPLIPAL2G160	no	no	no	b3825	DB02364,DB02983,DB04519,
3.1.1.5	R_LPLIPAL2G161	no	no	no	b3825	DB02364,DB02983,DB04519,
3.1.1.5	R_LPLIPAL2G180	no	no	no	b3825	DB02364,DB02983,DB04519,
3.1.1.5	R_LPLIPAL2G181	no	no	no	b3825	DB02364,DB02983,DB04519,
	R_LSERDHr	no	no	no	b1539	-
	R_LYSabcpp	no	no	no	(b2310 and b2307 and b2306 and b2308)	-
	R_LYSt2pp	no	no	no	b2156	-
	R_LYSt3pp	no	no	no	b2923	-
	R_LYXI	no	no	no	b3903	-
	R_LYXt2pp	no	no	no	b3907	-
1.1.1.17	R_M1PD	no	no	no	b3600	-
	R_MACPD	yes	no	no	b2323	-
1.1.1.83	R_MALDDH	no	no	no	b1800	-
	R_MALDt2_2pp	no	no	no	b3528	-
	R_MALt2_2pp	no	no	no	b3528	-
	R_MALTabcpp	no	no	no	(b4034 and b4033 and b4032 and b4035)	-
2.3.1.79	R_MALTATr	no	no	no	b0459	-
	R_MALTHXabcpp	no	no	no	(b4034 and b4033 and b4032 and b4035)	-
	R_MALTHXtexi	no	no	no	b4036	-
	R_MALTPTabcpp	no	no	no	(b4034 and b4033 and b4032 and b4035)	-
	R_MALTptspp	no	no	no	(b2417 and b1621 and b2415 and b2416)	-
	R_MALTPTtexi	no	no	no	b4036	-
	R_MALTtexi	no	no	no	b4036	-
	R_MALTTRabcpp	no	no	no	(b4034 and b4033 and b4032 and b4035)	-
	R_MALTTRtexi	no	no	no	b4036	-
	R_MALTTTRabcpp	no	no	no	(b4034 and b4033 and b4032 and b4035)	-
	R_MALTTTRtexi	no	no	no	b4036	-
2.7.7.22	R_MAN1PT2	no	no	no	b2049	-
5.3.1.8	R_MAN6PI	no	no	no	b1613	DB02171,DB03042,DB04184,
	R_MAN6Pt6_2pp	no	no	no	b3666	-
1.1.1.57	R_MANAO	no	no	no	b4323	-
	R_MANGLYCptspp	no	no	no	(b0731 and b2415 and b2416)	-

	R_MANPGH	no	no	no	b0732	-
	R_MANptspp	no	no	no	(b1817 and b1818 and b1819 and b2415 and b2416)	-
4.2.1.79	R_MCITD	no	no	no	b0334	-
4.1.3.30	R_MCITL2	no	no	no	b0331	-
4.1.3.31	R_MCITS	no	no	no	b0333	-
2.3.1.39	R_MCOATA	yes	yes	no	(b1094 and b1092)	-
2.8.1.2	R_MCPST	no	no	no	b2521	DB03352,
1.1.1.37	R_MDH	no	no	no	b3236	DB00336,DB02483,DB03461,
1.1.99.16	R_MDH2	no	no	no	b2210	-
1.1.99.16	R_MDH3	no	no	no	b2210	-
1.1.1.38	R_ME1	no	no	no	b1479	DB03680,
1.1.1.40	R_ME2	no	no	no	b2463	DB03461,
	R_MECDPDH2	yes	yes	yes	b2515	-
	R_MECDPS	yes	yes	no	b2746	-
	R_MELIBt2pp	no	no	no	b4120	-
	R_MELIBt3ipp	no	no	no	b1528	-
	R_MEPCT	yes	yes	yes	b2747	-
	R_METabcpp	no	no	no	(b0198 and b0199 and b0197)	-
2.5.1.6	R_METAT	yes	yes	yes	b2942	DB02212,DB03075,DB03191,DB03611,DB04554,
	R_METDabcpp	no	no	no	(b0198 and b0199 and b0197)	-
	R_METOX1s	no	no	no	s0001	-
	R_METOX2s	no	no	no	s0001	-
1.8.4.5	R_METSOXR1	no	no	no	((b4219 and b2582) or (b4219 and b3781))	-
	R_METSOXR2	no	no	no	((b1778 and b2582) or (b1778 and b3781))	-
6.1.1.10	R_METTRS	yes	no	yes	b2114	DB02151,DB02229,DB03799,DB03816,DB04015,
	R_MG2tpp	no	no	no	b3816	-
	R_MG2uabcpp	no	no	no	b4242	-
4.2.3.3	R_MGSA	no	no	no	b0963	DB03026,
3.1.3.25	R_MI1PP	yes	no	no	b2533	DB01356,DB04493,
4.2.1.99	R_MICITD	no	no	no	b0118	-
	R_MLTG1	no	no	no	b0403	-
	R_MLTG2	no	no	no	b0403	-
	R_MLTG3	no	no	no	b0403	-
	R_MLTG4	no	no	no	b0403	-
	R_MLTG5	no	no	no	b0403	-
	R_MLTP1	no	no	no	b3417	-
	R_MLTP2	no	no	no	b3417	-
	R_MLTP3	no	no	no	b3417	-
4.1.1.41	R_MMCD	no	no	no	b2919	-
	R_MMETt2pp	no	no	no	b0260	-
5.4.99.2	R_MMM2	no	no	no	b2917	-
	R_MN2tpp	no	no	no	b3040	-
	R_MN6PP	no	no	no	b0822	-
	R_MNLptspp	no	no	no	(b3599 and b2415 and b2416)	-
4.2.1.8	R_MNNH	no	no	no	b4322	-
	R_MNt2pp	no	no	no	b2392	-
	R_MOAT	yes	yes	yes	b3633	-
	R_MOAT2	yes	yes	yes	b3633	-
	R_MOAT3C	no	no	no	b3624	-
2.1.2.11	R_MOHMT	no	yes	no	b0134	-

	R_MSO3abcpp	no	no	no	(b0936 and b0933 and b0934)	-
3.2.2.16	R_MTAN	no	no	no	b0159	-
3.5.4.9	R_MTHFC	yes	yes	yes	b0529	-
1.5.1.5	R_MTHFD	yes	yes	no	b0529	-
1.5.1.20	R_MTHFR2	no	yes	no	b3941	-
1.5.3.2	R_MTRPOX	no	no	no	b1059	-
	R_N2Otpp	no	no	no	s0001	-
	R_NACODA	no	no	no	b3957	-
1.6.5.3	R_NADH10	no	no	no	b1109	-
1.6.5.3	R_NADH16pp	no	no	no	(b2276 and b2277 and b2278 and b2279 and b2280 and b2281 and b2282 and b2283 and b2284 and b2285 and b2286 and b2287 and b2288)	-
1.6.5.3	R_NADH17pp	no	no	no	(b2276 and b2277 and b2278 and b2279 and b2280 and b2281 and b2282 and b2283 and b2284 and b2285 and b2286 and b2287 and b2288)	-
1.6.5.3	R_NADH18pp	no	no	no	(b2276 and b2277 and b2278 and b2279 and b2280 and b2281 and b2282 and b2283 and b2284 and b2285 and b2286 and b2287 and b2288)	-
1.6.5.3	R_NADH5	no	no	no	b1109	-
1.6.5.3	R_NADH9	no	no	no	b1109	-
2.7.1.23	R_NADK	yes	yes	no	b2615	DB02212,DB03461,
1.6.99.6	R_NADPHQR2	no	no	no	b3028	-
1.6.99.6	R_NADPHQR3	no	no	no	b3028	-
1.6.99.6	R_NADPHQR4	no	no	no	b3028	-
6.3.1.5	R_NADS1	yes	yes	no	b1740	DB02212,DB02596,DB02937,
2.4.2.11	R_NAMNPP	no	no	no	b0931	DB03814,
	R_NAt3_1p5pp	no	no	no	b1186	-
	R_NAt3_2pp	no	no	no	b0019	-
	R_NHFRBO	no	no	no	(b2710 and b2711)	-
	R_NI2abcpp	no	no	no	b3469	-
	R_NI2tpp	no	no	no	b3816	-
	R_NMNPtpp	no	no	no	b0751	-
3.5.1.19	R_NNAM	no	no	no	b1768	-
2.7.7.18	R_NNATr	yes	yes	yes	b0639	-
	R_NNDMBRT	no	no	no	b1991	-
2.4.2.19	R_NNDPR	no	yes	no	b0109	DB02382,DB04294,
1.7.99.4	R_NO3R1bpp	no	no	no	((b2203 and b2206) and b2202 and b2205 and b2204)	-
1.7.99.4	R_NO3R2bpp	no	no	no	((b2203 and b2206) and b2202)	-

1.14.12.17	R_NODOx	no	no	no	b2552	DB03014,DB03979,
1.14.12.17	R_NODOy	no	no	no	b2552	DB03014,DB03979,
	R_NOtpp	no	no	no	s0001	-
4.1.3.36	R_NPHS	no	no	no	b2262	-
3.1.3.5	R_NTD10	no	no	no	b2744	DB00811,
3.1.3.5	R_NTD11	no	no	no	b2744	DB00811,
3.1.3.5	R_NTD4	no	no	no	b2744	DB00811,
3.1.3.5	R_NTD7	no	no	no	b2744	DB00811,
3.1.3.5	R_NTD9	no	no	no	b2744	DB00811,
3.6.1.15	R_NTP11	no	no	no	b4394	DB01720,DB02331,DB03388,DB03605,DB03647,DB04298,
3.6.1.15	R_NTP12	no	no	no	b4394	DB01720,DB02331,DB03388,DB03605,DB03647,DB04298,
3.6.1.15	R_NTP3	no	no	no	b4161	DB01720,DB02331,DB03388,DB03605,DB03647,DB04298,
3.6.1.15	R_NTP3pp	no	no	no	b0980	DB01720,DB02331,DB03388,DB03605,DB03647,DB04298,
3.6.1.15	R_NTP5	no	no	no	b4161	DB01720,DB02331,DB03388,DB03605,DB03647,DB04298,
3.6.1.19	R_NTPP10	no	no	no	b2954	-
3.6.1.19	R_NTPP11	no	no	no	b2954	-
	R_NTPP6	no	no	no	b2781	-
	R_NTPP8	no	no	no	b2781	-
3.6.1.19	R_NTPP9	no	no	no	b2954	-
3.1.5.1	R_NTPTP1	no	no	no	b0160	-
3.1.5.1	R_NTPTP2	no	no	no	b0160	-
	R_NTRIR2x	no	no	no	(b3365 and b3366)	-
	R_NTRIR3pp	no	no	no	(b4070 and b4071 and b4072 and b4073)	-
	R_NTRIR4pp	no	no	no	(b4070 and b4071 and b4072 and b4073)	-
	R_O16A4Lpp	no	no	no	b3622	-
	R_O16AP1pp	no	no	no	(b2035 and b2027)	-
	R_O16AP2pp	no	no	no	(b2035 and b2027)	-
	R_O16AP3pp	no	no	no	(b2035 and b2027)	-
	R_O16AT	no	no	no	b2033	-
	R_O16AUNDtpp	no	no	no	b2037	-
	R_O16GALFT	no	no	no	b2034	-
	R_O16GLCT1	no	no	no	b2032	-
	R_O2tpp	no	yes	no	s0001	-
4.1.1.3	R_OAADC	no	no	no	b1850	-
	R_OBTFL	no	no	no	(((b0902 and b0903) and b2579) or (b0902 and b0903) or (b0902 and b3114))	-
	R_OCDCAtexi	no	no	no	b2344	-
	R_OCDCEAtexi	no	no	no	b2344	-
	R_OCTDPS	yes	yes	yes	b3187	-
5.3.3.8	R_ODECOAI	no	no	no	b3846	-
2.6.1.52	R_OHPBAT	no	yes	yes	b0907	DB02327,
	R_OHPHM	no	no	no	b2232	-
	R_OMBZLM	no	no	no	b3833	-
	R_OMCDC	no	yes	no	b0073	-
	R_OMMBLHX	no	no	no	b0662	-
4.1.1.23	R_OMPDC	no	yes	no	b1281	DB01915,DB02202,DB02890,DB02957,DB03668,DB03718,

	R_OMPHHX	no	no	no	b2907	-
4.2.1.80	R_OP4ENH	no	no	no	b0350	-
	R_OPHHX	yes	no	no	b3835	-
	R_ORNabcpp	no	no	no	(b2310 and b2307 and b2306 and b2308)	-
	R_OROTt2_2pp	no	no	no	b3528	-
2.4.2.10	R_ORPT	no	yes	yes	b3642	DB01632,
4.1.1.71	R_OXGDC2	no	no	no	b2264	-
1.5.1.12	R_P5CD	no	no	no	b1014	DB03051,DB04184,
1.5.1.2	R_P5CR	no	yes	no	b0386	-
	R_PA120abcpp	yes	no	yes	b0914	-
	R_PA140abcpp	yes	no	yes	b0914	-
	R_PA141abcpp	yes	no	yes	b0914	-
	R_PA160abcpp	yes	no	yes	b0914	-
	R_PA161abcpp	yes	no	yes	b0914	-
	R_PA180abcpp	yes	no	yes	b0914	-
	R_PA181abcpp	yes	no	yes	b0914	-
6.2.1.30	R_PACCOAL	no	no	no	b1398	-
6.3.2.1	R_PANTS	no	yes	yes	b0133	DB02596,
3.1.3.4	R_PAPA120	no	no	no	b1278	-
3.1.3.4	R_PAPA120pp	no	no	no	b1278	-
3.1.3.4	R_PAPA140	no	no	no	b1278	-
3.1.3.4	R_PAPA140pp	no	no	no	b1278	-
3.1.3.4	R_PAPA141	no	no	no	b1278	-
3.1.3.4	R_PAPA141pp	no	no	no	b1278	-
3.1.3.4	R_PAPA160	no	no	no	b1278	-
3.1.3.4	R_PAPA160pp	no	no	no	b1278	-
3.1.3.4	R_PAPA161	no	no	no	b1278	-
3.1.3.4	R_PAPA161pp	no	no	no	b1278	-
3.1.3.4	R_PAPA180	no	no	no	b1278	-
3.1.3.4	R_PAPA180pp	no	no	no	b1278	-
3.1.3.4	R_PAPA181	no	no	no	b1278	-
3.1.3.4	R_PAPA181pp	no	no	no	b1278	-
2.7.8.13	R_PAPPT3	yes	yes	yes	b0087	-
1.8.4.8	R_PAPSR	no	no	no	((b2762 and b2582) or (b2762 and b3781))	-
1.8.4.8	R_PAPSR2	no	no	no	((b2762 and b0849) or (b2762 and b1064) or (b2762 and b1654) or (b2762 and b3610))	-
3.1.4.17	R_PDE1	no	no	no	b1489	DB01640,DB01647,DB01791,DB01954,DB01959,DB01970,DB02660,DB02676,DB02918,DB03183,DB03349,DB03606,DB03807,DB03849,DB04149,DB04271,DB04469,DB04530,
	R_PDH	no	no	no	(b0114 and b0115 and b0116)	-
1.4.3.5	R_PDX5POi	no	no	no	b1638	-
	R_PDX5PS	no	yes	no	(b0052 and b2564)	-
	R_PE120abcpp	yes	no	yes	b0914	-
	R_PE140abcpp	yes	no	yes	b0914	-
	R_PE141abcpp	yes	no	yes	b0914	-
	R_PE160abcpp	yes	yes	yes	b0914	-
	R_PE161abcpp	yes	yes	yes	b0914	-
	R_PE180abcpp	yes	no	yes	b0914	-
	R_PE181abcpp	yes	no	yes	b0914	-

1.4.3.6	R_PEAMNOpp	no	no	no	b1386	DB01634,DB01657,DB02511,DB02537,DB02928,DB03631,DB04334,
	R_PERD	no	yes	no	b2320	-
	R_PETNT161pp	no	no	no	b3546	-
	R_PETNT181pp	no	no	no	b3546	-
2.7.1.11	R_PFK_2	no	no	no	b3916	DB04493,
	R_PG120abcpp	yes	no	yes	b0914	-
	R_PG140abcpp	yes	no	yes	b0914	-
	R_PG141abcpp	yes	no	yes	b0914	-
	R_PG160abcpp	yes	no	yes	b0914	-
	R_PG161abcpp	yes	no	yes	b0914	-
	R_PG180abcpp	yes	no	yes	b0914	-
	R_PG181abcpp	yes	no	yes	b0914	-
5.4.2.10	R_PGAMT	no	yes	no	b3176	-
1.1.1.95	R_PGCD	no	no	no	b2913	DB03806,
5.3.1.9	R_PGI	no	no	no	b4025	DB02093,DB02171,DB02257,DB03042,DB03937,DB04493,
2.7.2.3	R_PGK	yes	no	no	b2926	-
3.1.1.31	R_PGL	no	no	no	b0767	DB03754,
3.1.3.18	R_PGLYCP	no	no	no	b3385	DB04184,
	R_PGP120abcpp	yes	no	yes	b0914	-
	R_PGP140abcpp	yes	no	yes	b0914	-
	R_PGP141abcpp	yes	no	yes	b0914	-
	R_PGP160abcpp	yes	no	yes	b0914	-
	R_PGP161abcpp	yes	no	yes	b0914	-
	R_PGP180abcpp	yes	no	yes	b0914	-
	R_PGP181abcpp	yes	no	yes	b0914	-
2.7.8.5	R_PGSA120	yes	no	yes	b1912	-
2.7.8.5	R_PGSA140	yes	no	yes	b1912	-
2.7.8.5	R_PGSA141	yes	no	yes	b1912	-
2.7.8.5	R_PGSA160	yes	no	yes	b1912	-
2.7.8.5	R_PGSA161	yes	no	yes	b1912	-
2.7.8.5	R_PGSA180	yes	no	yes	b1912	-
2.7.8.5	R_PGSA181	yes	no	yes	b1912	-
	R_PHEMEabcpp	no	no	no	(b2201 and b2200 and b2199)	-
6.1.1.20	R_PHETRS	yes	no	yes	(b1713 and b1714)	-
	R_PHYTSpp	no	no	no	b0980	-
	R_Pluabcpp	no	no	no	(b3726 and b3725 and b3727 and b3728)	-
3.1.1.32	R_PLIPA1A120pp	no	no	no	b3821	DB03122,DB03692,
3.1.1.32	R_PLIPA1A140pp	no	no	no	b3821	DB03122,DB03692,
3.1.1.32	R_PLIPA1A141pp	no	no	no	b3821	DB03122,DB03692,
3.1.1.32	R_PLIPA1A160pp	no	no	no	b3821	DB03122,DB03692,
3.1.1.32	R_PLIPA1A161pp	no	no	no	b3821	DB03122,DB03692,
3.1.1.32	R_PLIPA1A180pp	no	no	no	b3821	DB03122,DB03692,
3.1.1.32	R_PLIPA1A181pp	no	no	no	b3821	DB03122,DB03692,
3.1.1.32	R_PLIPA1E120pp	no	no	no	b3821	DB03122,DB03692,
3.1.1.32	R_PLIPA1E140pp	no	no	no	b3821	DB03122,DB03692,
3.1.1.32	R_PLIPA1E141pp	no	no	no	b3821	DB03122,DB03692,
3.1.1.32	R_PLIPA1E160pp	no	no	no	b3821	DB03122,DB03692,
3.1.1.32	R_PLIPA1E161pp	no	no	no	b3821	DB03122,DB03692,
3.1.1.32	R_PLIPA1E180pp	no	no	no	b3821	DB03122,DB03692,
3.1.1.32	R_PLIPA1E181pp	no	no	no	b3821	DB03122,DB03692,
3.1.1.32	R_PLIPA1G120pp	no	no	no	b3821	DB03122,DB03692,
3.1.1.32	R_PLIPA1G140pp	no	no	no	b3821	DB03122,DB03692,
3.1.1.32	R_PLIPA1G141pp	no	no	no	b3821	DB03122,DB03692,
3.1.1.32	R_PLIPA1G160pp	no	no	no	b3821	DB03122,DB03692,
3.1.1.32	R_PLIPA1G161pp	no	no	no	b3821	DB03122,DB03692,
3.1.1.32	R_PLIPA1G180pp	no	no	no	b3821	DB03122,DB03692,

3.1.1.32	R_PLIPA1G181pp	no	no	no	b3821	DB03122,DB03692,
3.1.1.4	R_PLIPA2A120pp	no	no	no	b3821	DB00233,DB01103,DB01955,DB02210,DB02448,DB02504,DB02636,DB02936,DB02938,DB03121,DB03122,DB03471,DB03565,DB03585,DB03587,DB03633,DB03692,DB03784,DB04077,DB04112,DB04184,DB04287,DB04402,DB04743,
3.1.1.4	R_PLIPA2A140pp	no	no	no	b3821	DB00233,DB01103,DB01955,DB02210,DB02448,DB02504,DB02636,DB02936,DB02938,DB03121,DB03122,DB03471,DB03565,DB03585,DB03587,DB03633,DB03692,DB03784,DB04077,DB04112,DB04184,DB04287,DB04402,DB04743,
3.1.1.4	R_PLIPA2A141pp	no	no	no	b3821	DB00233,DB01103,DB01955,DB02210,DB02448,DB02504,DB02636,DB02936,DB02938,DB03121,DB03122,DB03471,DB03565,DB03585,DB03587,DB03633,DB03692,DB03784,DB04077,DB04112,DB04184,DB04287,DB04402,DB04743,
3.1.1.4	R_PLIPA2A160pp	no	no	no	b3821	DB00233,DB01103,DB01955,DB02210,DB02448,DB02504,DB02636,DB02936,DB02938,DB03121,DB03122,DB03471,DB03565,DB03585,DB03587,DB03633,DB03692,DB03784,DB04077,DB04112,DB04184,DB04287,DB04402,DB04743,
3.1.1.4	R_PLIPA2A161pp	no	no	no	b3821	DB00233,DB01103,DB01955,DB02210,DB02448,DB02504,DB02636,DB02936,DB02938,DB03121,DB03122,DB03471,DB03565,DB03585,DB03587,DB03633,DB03692,DB03784,DB04077,DB04112,DB04184,DB04287,DB04402,DB04743,
3.1.1.4	R_PLIPA2A180pp	no	no	no	b3821	DB00233,DB01103,DB01955,DB02210,DB02448,DB02504,DB02636,DB02936,DB02938,DB03121,DB03122,DB03471,DB03565,DB03585,DB03587,DB03633,DB03692,DB03784,DB04077,DB04112,DB04184,DB04287,DB04402,DB04743,
3.1.1.4	R_PLIPA2A181pp	no	no	no	b3821	DB00233,DB01103,DB01955,DB02210,DB02448,DB02504,DB02636,DB02936,DB02938,DB03121,DB03122,DB03471,DB03565,DB03585,DB03587,DB03633,DB03692,DB03784,DB04077,DB04112,DB04184,DB04287,DB04402,DB04743,

3.1.1.4	R_PLIPA2E120pp	no	no	no	b3821	DB00233,DB01103,DB01955,DB02210,DB02448,DB02504,DB02636,DB02936,DB02938,DB03121,DB03122,DB03471,DB03565,DB03585,DB03587,DB03633,DB03692,DB03784,DB04077,DB04112,DB04184,DB04287,DB04402,DB04743,
3.1.1.4	R_PLIPA2E140pp	no	no	no	b3821	DB00233,DB01103,DB01955,DB02210,DB02448,DB02504,DB02636,DB02936,DB02938,DB03121,DB03122,DB03471,DB03565,DB03585,DB03587,DB03633,DB03692,DB03784,DB04077,DB04112,DB04184,DB04287,DB04402,DB04743,
3.1.1.4	R_PLIPA2E141pp	no	no	no	b3821	DB00233,DB01103,DB01955,DB02210,DB02448,DB02504,DB02636,DB02936,DB02938,DB03121,DB03122,DB03471,DB03565,DB03585,DB03587,DB03633,DB03692,DB03784,DB04077,DB04112,DB04184,DB04287,DB04402,DB04743,
3.1.1.4	R_PLIPA2E160pp	no	no	no	b3821	DB00233,DB01103,DB01955,DB02210,DB02448,DB02504,DB02636,DB02936,DB02938,DB03121,DB03122,DB03471,DB03565,DB03585,DB03587,DB03633,DB03692,DB03784,DB04077,DB04112,DB04184,DB04287,DB04402,DB04743,
3.1.1.4	R_PLIPA2E161pp	no	no	no	b3821	DB00233,DB01103,DB01955,DB02210,DB02448,DB02504,DB02636,DB02936,DB02938,DB03121,DB03122,DB03471,DB03565,DB03585,DB03587,DB03633,DB03692,DB03784,DB04077,DB04112,DB04184,DB04287,DB04402,DB04743,
3.1.1.4	R_PLIPA2E180pp	no	no	no	b3821	DB00233,DB01103,DB01955,DB02210,DB02448,DB02504,DB02636,DB02936,DB02938,DB03121,DB03122,DB03471,DB03565,DB03585,DB03587,DB03633,DB03692,DB03784,DB04077,DB04112,DB04184,DB04287,DB04402,DB04743,
3.1.1.4	R_PLIPA2E181pp	no	no	no	b3821	DB00233,DB01103,DB01955,DB02210,DB02448,DB02504,DB02636,DB02936,DB02938,DB03121,DB03122,DB03471,DB03565,DB03585,DB03587,DB03633,DB03692,DB03784,DB04077,DB04112,DB04184,DB04287,DB04402,DB04743,

3.1.1.4	R_PLIPA2G120pp	no	no	no	b3821	DB00233,DB01103,DB01955,DB02210,DB02448,DB02504,DB02636,DB02936,DB02938,DB03121,DB03122,DB03471,DB03565,DB03585,DB03587,DB03633,DB03692,DB03784,DB04077,DB04112,DB04184,DB04287,DB04402,DB04743,
3.1.1.4	R_PLIPA2G140pp	no	no	no	b3821	DB00233,DB01103,DB01955,DB02210,DB02448,DB02504,DB02636,DB02936,DB02938,DB03121,DB03122,DB03471,DB03565,DB03585,DB03587,DB03633,DB03692,DB03784,DB04077,DB04112,DB04184,DB04287,DB04402,DB04743,
3.1.1.4	R_PLIPA2G141pp	no	no	no	b3821	DB00233,DB01103,DB01955,DB02210,DB02448,DB02504,DB02636,DB02936,DB02938,DB03121,DB03122,DB03471,DB03565,DB03585,DB03587,DB03633,DB03692,DB03784,DB04077,DB04112,DB04184,DB04287,DB04402,DB04743,
3.1.1.4	R_PLIPA2G160pp	no	no	no	b3821	DB00233,DB01103,DB01955,DB02210,DB02448,DB02504,DB02636,DB02936,DB02938,DB03121,DB03122,DB03471,DB03565,DB03585,DB03587,DB03633,DB03692,DB03784,DB04077,DB04112,DB04184,DB04287,DB04402,DB04743,
3.1.1.4	R_PLIPA2G161pp	no	no	no	b3821	DB00233,DB01103,DB01955,DB02210,DB02448,DB02504,DB02636,DB02936,DB02938,DB03121,DB03122,DB03471,DB03565,DB03585,DB03587,DB03633,DB03692,DB03784,DB04077,DB04112,DB04184,DB04287,DB04402,DB04743,
3.1.1.4	R_PLIPA2G180pp	no	no	no	b3821	DB00233,DB01103,DB01955,DB02210,DB02448,DB02504,DB02636,DB02936,DB02938,DB03121,DB03122,DB03471,DB03565,DB03585,DB03587,DB03633,DB03692,DB03784,DB04077,DB04112,DB04184,DB04287,DB04402,DB04743,
3.1.1.4	R_PLIPA2G181pp	no	no	no	b3821	DB00233,DB01103,DB01955,DB02210,DB02448,DB02504,DB02636,DB02936,DB02938,DB03121,DB03122,DB03471,DB03565,DB03585,DB03587,DB03633,DB03692,DB03784,DB04077,DB04112,DB04184,DB04287,DB04402,DB04743,
5.4.2.8	R_PMANM	no	no	no	b2048	DB04522,
2.7.4.7	R_PMPK	no	yes	no	b2103	-
2.7.1.33	R_PNTK	no	yes	no	b3974	DB01783,
	R_PNTOt4pp	no	no	no	b3258	-
1.2.2.2	R_POX	no	no	no	b0871	DB00336,
2.7.2.1	R_PPAKr	no	no	no	b3115	DB02423,
	R_PPAt4pp	no	no	no	b1015	-

4.2.1.24	R_PPBNGS	yes	yes	no	b0369	DB02068,DB02239,DB02260,DB02828,DB02878,DB04344,DB04530,DB04560,
4.1.1.31	R_PPC	no	yes	no	b3956	DB04317,
4.1.1.36	R_PPCDC	yes	yes	no	b3639	-
4.1.1.49	R_PPCK	no	no	no	b3403	DB04184,
	R_PPCSCT	no	no	no	b2920	-
3.1.7.2	R_PPGPPDP	yes	no	no	b3650	-
2.7.4.1	R_PPK2r	no	no	no	b2501	-
2.7.4.1	R_PPKr	no	no	yes	b2501	-
5.4.2.7	R_PPM2	no	no	no	b4383	-
6.3.2.5	R_PPNCL2	yes	yes	no	b3639	-
1.3.1.12	R_PPND	no	yes	no	b2600	-
4.2.1.51	R_PPNDH	no	yes	no	b2599	-
1.3.3.4	R_PPPGO	yes	no	no	b3850	DB03272,
1.3.3.4	R_PPPGO3	yes	no	no	b3850	DB03272,
	R_PPPNDO	no	no	no	(b2538 and b2539 and b2540 and b2542)	-
	R_PPPNt2rpp	no	no	no	b2536	-
2.7.9.2	R_PPS	no	no	no	b1702	-
	R_PPTHpp	no	no	no	b0383	-
6.3.4.13	R_PRAGSr	no	yes	no	b4005	DB03546,
5.3.1.24	R_PRAIi	no	yes	no	b1262	DB03543,
6.3.3.1	R_PRAIS	no	yes	no	b2499	DB03546,
3.5.4.19	R_PRAMPC	no	yes	no	b2026	-
6.3.2.6	R_PRASCSi	no	yes	no	b2476	-
3.6.1.31	R_PRATPP	no	yes	no	b2026	-
6.3.5.3	R_PRFGS	no	yes	no	b2557	-
5.3.1.16	R_PRMICI	no	yes	no	b2024	-
	R_PROabcpp	no	no	no	(b2677 and b2678 and b2679)	-
1.5.99.8	R_PROD2	no	no	no	b1014	DB03051,DB04184,
	R_PROGLYabcpp	no	no	no	(b3544 and b3543 and b3542 and b3541 and b3540)	-
	R_PROt2rpp	no	no	no	b4111	-
	R_PROt4pp	no	no	no	b1015	-
6.1.1.15	R_PROTRS	yes	no	yes	b0194	DB02510,DB03376,
2.7.6.1	R_PRPPS	yes	no	no	b1207	DB02798,DB03148,
	R_PSCLYSt2pp	no	no	no	b3370	-
2.5.1.19	R_PSCVT	no	yes	yes	b0908	DB02592,DB03116,
4.1.1.65	R_PSD120	yes	no	yes	b4160	-
4.1.1.65	R_PSD140	yes	no	yes	b4160	-
4.1.1.65	R_PSD141	yes	no	yes	b4160	-
4.1.1.65	R_PSD160	yes	yes	yes	b4160	-
4.1.1.65	R_PSD161	yes	yes	yes	b4160	-
4.1.1.65	R_PSD180	yes	no	yes	b4160	-
4.1.1.65	R_PSD181	yes	no	yes	b4160	-
2.6.1.52	R_PSERT	no	no	no	b0907	DB02327,
3.1.3.3	R_PSP_L	no	no	no	b4388	DB03292,DB04156,DB04444,DB04522,
	R_PSP_Lpp	no	no	no	b4055	-
2.7.8.8	R_PSSA120	yes	no	yes	b2585	-
2.7.8.8	R_PSSA140	yes	no	yes	b2585	-
2.7.8.8	R_PSSA141	yes	no	yes	b2585	-
2.7.8.8	R_PSSA160	yes	yes	yes	b2585	-
2.7.8.8	R_PSSA161	yes	yes	yes	b2585	-
2.7.8.8	R_PSSA180	yes	no	yes	b2585	-
2.7.8.8	R_PSSA181	yes	no	yes	b2585	-
	R_PTA2	no	no	no	b2297	-
	R_PTHRpp	no	no	no	b4055	-
2.7.7.3	R_PTPATi	yes	yes	yes	b3634	-

	R_PTRCORNt7pp	no	no	no	b0692	-
2.6.1.29	R_PTRCTA	no	no	no	b3073	-
2.4.2.1	R_PUNP1	no	no	no	b4384	DB00194,DB00787,DB01667,DB02066,DB02113,DB02222,DB02230,DB02391,DB02392,DB02568,DB02796,DB02896,DB02934,DB02947,DB02985,DB03172,DB03411,DB03528,DB03551,DB03609,DB03735,DB03743,DB03952,DB03986,DB04095,DB04198,DB04260,DB04441,DB04753,DB04754,DB04757,
2.4.2.1	R_PUNP2	no	no	no	b4384	DB00194,DB00787,DB01667,DB02066,DB02113,DB02222,DB02230,DB02391,DB02392,DB02568,DB02796,DB02896,DB02934,DB02947,DB02985,DB03172,DB03411,DB03528,DB03551,DB03609,DB03735,DB03743,DB03952,DB03986,DB04095,DB04198,DB04260,DB04441,DB04753,DB04754,DB04757,
2.4.2.1	R_PUNP7	no	no	no	b2407	DB00194,DB00787,DB01667,DB02066,DB02113,DB02222,DB02230,DB02391,DB02392,DB02568,DB02796,DB02896,DB02934,DB02947,DB02985,DB03172,DB03411,DB03528,DB03551,DB03609,DB03735,DB03743,DB03952,DB03986,DB04095,DB04198,DB04260,DB04441,DB04753,DB04754,DB04757,
1.4.3.5	R_PYAM5PO	no	no	no	b1638	-
2.7.1.35	R_PYDAMK	no	no	no	b2418	DB04770,DB04776,
2.7.1.35	R_PYDXNK	no	no	no	b2418	DB04770,DB04776,
2.4.2.2	R_PYNP2r	no	no	no	b3831	-
	R_QMO2	no	no	no	b3029	-
	R_QMO3	no	no	no	b3029	-
	R_QULNS	no	yes	no	b0750	-
	R_R15BPK	no	no	no	b4094	-
	R_R5PP	no	no	no	b0822	-
	R_R5PPpp	no	no	no	b4055	-
2.7.1.26	R_RBFK	yes	yes	yes	b0025	-
2.5.1.9	R_RBFSa	yes	yes	no	b1662	DB02135,DB02184,DB02214,DB02290,DB02693,DB02711,DB03022,DB03812,DB03883,DB03973,DB04128,DB04162,DB04262,DB04266,
2.5.1.9	R_RBFSb	yes	yes	no	b0415	DB02135,DB02184,DB02214,DB02290,DB02693,DB02711,DB03022,DB03812,DB03883,DB03973,DB04128,DB04162,DB04262,DB04266,
2.7.1.15	R_RBK	no	no	no	b3752	DB01936,DB04444,
2.7.1.16	R_RBK_L1	no	no	no	b0063	-
	R_RHAT1	no	no	no	b3629	-
	R_RHCCE	no	yes	no	b2687	-
5.3.1.14	R_RMI	no	no	no	b3903	-
2.7.1.5	R_RMK	no	no	no	b3904	-
	R_RMNtpp	no	no	no	b3907	-
4.1.2.19	R_RMPA	no	no	no	b3902	DB03026,

1.17.4.1	R_RNDR1	yes	no	yes	(((b2234 and b2235) and b3781) or ((b2234 and b2235) and b2582))	DB02452,DB04077,
	R_RNDR1b	no	no	no	((b1654 and (b2675 and b2676)) or (b0849 and (b2675 and b2676)) or (b1064 and (b2675 and b2676)) or (b3610 and (b2675 and b2676)))	-
1.17.4.1	R_RNDR2	yes	no	yes	(((b2234 and b2235) and b2582) or ((b2234 and b2235) and b3781))	DB02452,DB04077,
	R_RNDR2b	no	no	no	((b3610 and (b2675 and b2676)) or (b1064 and (b2675 and b2676)) or (b1654 and (b2675 and b2676)) or (b0849 and (b2675 and b2676)))	-
1.17.4.1	R_RNDR3	yes	no	yes	(((b2234 and b2235) and b2582) or ((b2234 and b2235) and b3781))	DB02452,DB04077,
	R_RNDR3b	no	no	no	((b1654 and (b2675 and b2676)) or (b1064 and (b2675 and b2676)) or (b0849 and (b2675 and b2676)) or (b3610 and (b2675 and b2676)))	-
1.17.4.1	R_RNDR4	yes	no	yes	(((b2234 and b2235) and b2582) or ((b2234 and b2235) and b3781))	DB02452,DB04077,
	R_RNDR4b	no	no	no	((b1064 and (b2675 and b2676)) or (b3610 and (b2675 and b2676)) or (b1654 and (b2675 and b2676)) or (b0849 and (b2675 and b2676)))	-

	R_RNTR1c	no	no	no	((b2895 and b3924 and b4238 and b4237) or (b2895 and b4238) or (b0684 and b4238) or (b0684 and b3924 and b4238 and b4237))	-
	R_RNTR2c	no	no	no	((b0684 and b4238) or (b2895 and b4238) or (b2895 and b3924 and b4238 and b4237) or (b0684 and b3924 and b4238 and b4237))	-
	R_RNTR3c	no	no	no	((b2895 and b4238) or (b0684 and b3924 and b4238 and b4237) or (b0684 and b4238) or (b2895 and b3924 and b4238 and b4237))	-
	R_RNTR4c	no	no	no	((b0684 and b3924 and b4238 and b4237) or (b2895 and b4238) or (b0684 and b4238) or (b2895 and b3924 and b4238 and b4237))	-
	R_RZ5PP	no	no	no	b0638	-
	R_S7PI	no	no	no	b0222	-
2.6.1.69	R_SADH	no	no	no	b1745	-
	R_SADT2	no	yes	no	(b2751 and b2752)	-
1.5.3.1	R_SARCOX	no	no	no	b1059	DB01918,DB03098,DB03517,
1.1.1.140	R_SBTPD	no	no	no	b2705	-
	R_SBTptspp	no	no	no	(b2415 and b2416 and b2702 and b2704 and b2703)	-
3.5.1.18	R_SDPDS	yes	yes	yes	b2472	-
2.6.1.17	R_SDPTA	no	yes	no	b3359	-
2.9.1.1	R_SELCYSS	no	no	no	b3591	-
2.7.9.3	R_SELNPS	no	no	no	b1764	-
	R_SERASr	no	no	no	b0586	-
2.3.1.30	R_SERAT	no	yes	no	b3607	-
	R_SERD_D	no	no	no	b2366	-
	R_SERt4pp	no	no	no	b3089	-
6.1.1.11	R_SERTRS	yes	no	yes	b0893	-
6.1.1.11	R_SERTRS2	yes	no	yes	b0893	-
	R_SGDS	no	no	no	b1744	-
	R_SGSAD	no	no	no	b1746	-
	R_SHCHCS2	no	no	no	b2264	-
	R_SHCHD2	no	yes	no	b3368	-
	R_SHCHF	no	yes	yes	b3368	-
4.2.99.9	R_SHSL1	no	yes	yes	b3939	-
	R_SKMt2pp	no	no	no	b1981	-

	R_SO2tpp	no	no	yes	s0001	-
	R_SOTA	no	no	no	b1748	-
2.3.1.57	R_SPMDAT1	no	no	no	b1584	-
2.3.1.57	R_SPMDAT2	no	no	no	b1584	-
2.5.1.16	R_SPMS	no	no	no	b0121	DB02844,
1.15.1.1	R_SPODMpp	no	no	no	b1646	DB03297,DB03382,DB04184,DB04436,
1.2.1.16	R_SSALy	no	no	no	b2661	-
6.2.1.26	R_SUCBZL	no	no	no	b2260	-
	R_SUCBZS	no	no	no	b2261	-
	R_SUCCt2_2pp	no	no	no	b3528	-
1.3.99.1	R_SUCDi	no	no	no	(b0721 and b0722 and b0723 and b0724)	DB00730,DB03014,DB03343,DB04631,
6.2.1.5	R_SUCOAS	no	no	no	(b0728 and b0729)	-
	R_SUCptspp	no	no	no	(b2417 and b2429 and b2415 and b2416)	-
	R_SULFACabcpp	no	no	no	(b0936 and b0933 and b0934)	-
1.8.2.2	R_SULRi	no	yes	no	(b2763 and b2764)	-
	R_T2DECAI	yes	yes	no	b0954	-
1.1.1.58	R_TAGURr	no	no	no	b1521	-
4.2.1.32	R_TARTD	no	no	no	(b3061 and b3062)	-
	R_TARTRt7pp	no	no	no	b3063	-
1.14.11.17	R_TAUDO	no	no	no	b0368	DB03806,
	R_TAURabcpp	no	no	no	(b0365 and b0366 and b0367)	-
5.3.3.8	R_TDECOAI	no	no	no	b3846	-
3.6.1.15	R_TDP	no	no	no	b1134	DB01720,DB02331,DB03388,DB03605,DB03647,DB04298,
	R_TDPADGAT	no	no	no	b3790	-
2.6.1.33	R_TDPAGTA	no	no	no	b3791	-
5.1.3.13	R_TDPDRE	no	no	no	b2038	DB02549,DB03161,DB03751,DB04530,
1.1.1.133	R_TDPDRR	no	no	no	b2040	-
2.7.1.130	R_TDSK	yes	yes	yes	b0915	-
	R_TDSR1	no	no	no	(b2893 and b4136)	-
	R_TDSR2	no	no	no	(b4136 and b0604)	-
1.6.1.1	R_THD2pp	no	no	no	(b1602 and b1603)	-
2.3.1.117	R_THDPS	yes	yes	no	b0166	DB03134,DB03905,
	R_THIORDXi	no	no	no	((b2480 and b2582) or (b2480 and b3781))	-
	R_THMabcpp	no	no	no	(b0068 and b0067 and b0066)	-
	R_THMDt2rpp	no	no	no	b2406	-
	R_THRabcpp	no	no	no	(b3454 and b3455 and b3457 and b3460 and b3456)	-
1.1.1.103	R_THRD	no	no	no	b3616	-
4.2.3.1	R_THRS	no	yes	no	b0004	-
	R_THRt2rpp	no	no	no	b3116	-
	R_THRt4pp	no	no	no	b3089	-
6.1.1.3	R_THRTRS	yes	no	yes	b1719	DB03355,DB03869,DB04024,

	R_THZPSN	no	yes	no	(b2530 and b3992 and (b3990 and b3991) and b0423 and b4407)	-
	R_TMAOR2	no	no	no	(b0894 and b0895 and b0896)	-
2.7.1.21	R_TMDK1	no	no	no	b1238	DB00194,DB00299,DB00432,DB00577,DB00787,DB01004,DB01730,DB02324,DB02452,DB02495,DB02500,DB02765,DB02921,DB03000,DB03280,DB03312,DB03778,DB03804,DB04139,DB04438,
2.4.2.4	R_TMDPP	no	no	yes	b4382	DB02631,
2.1.1.45	R_TMDS	no	yes	no	b2827	DB00432,DB01099,DB02223,DB02301,DB02467,DB02752,DB02899,DB03038,DB03157,DB03274,DB03541,DB03558,DB03761,DB03800,DB03818,DB04447,DB04503,DB04530,DB04696,
2.7.1.89	R_TMK	no	no	no	b1106	-
2.7.4.16	R_TMPK	yes	yes	no	b0417	-
2.5.1.3	R_TMPPP	no	yes	no	b3993	DB01788,DB02212,DB02254,DB02885,DB03145,
5.3.1.1	R_TPI	no	no	no	b3919	DB01695,DB01709,DB01779,DB02515,DB02764,DB03026,DB03132,DB03135,DB03314,DB03379,DB03900,DB04184,DB04326,DB04447,
	R_TPRDCOAS	no	no	no	b0613	-
3.2.1.93	R_TRE6PH	no	no	no	b4239	-
3.1.3.12	R_TRE6PP	no	no	no	b1897	-
2.4.1.15	R_TRE6PS	no	no	no	b1896	DB03488,DB04355,
3.2.1.28	R_TREH	no	no	no	b3519	-
3.2.1.28	R_TREHpp	no	no	no	b1197	-
	R_TREptspp	no	no	no	(b2417 and b2415 and b2416 and b4240)	-
4.1.99.1	R_TRPAS2	no	no	no	b3708	-
4.2.1.20	R_TRPS1	no	no	no	(b1260 and b1261)	DB03171,
4.2.1.20	R_TRPS2	no	no	no	(b1260 and b1261)	DB03171,
4.2.1.20	R_TRPS3	no	no	no	(b1260 and b1261)	DB03171,
6.1.1.2	R_TRPTRS	yes	no	yes	b3384	-
	R_TSULabcpp	no	no	no	((b2422 and b2425 and b2424 and b2423) or (b2422 and b2424 and b2423 and b3917))	-
	R_TTDCAtexi	no	no	no	b2344	-
	R_TTDCEAtexi	no	no	no	b2344	-
	R_TUNGSabcpp	no	no	no	(b0763 and b0764 and b0765)	-
1.4.3.6	R_TYROXDApp	no	no	no	b1386	DB01634,DB01657,DB02511,DB02537,DB02928,DB03631,DB04334,
	R_TYRPpp	no	no	no	b4055	-
6.1.1.1	R_TYRTRS	yes	no	yes	b1637	DB03325,DB03978,
	R_U23GAAT	yes	yes	yes	b0179	-
6.3.2.13	R_UAAGDS	yes	yes	yes	b0085	-

3.6.1.45	R_UACGALPpp	no	no	no	b0480	-
3.6.1.45	R_UACGAMPpp	no	no	no	b0480	-
	R_UACMAMO	no	no	no	b3787	-
5.1.3.14	R_UAG2E	no	no	no	b3786	DB02196,
2.3.1.129	R_UAGAAT	yes	yes	no	(b1094 and b0181)	-
2.5.1.7	R_UAGCVT	yes	yes	yes	b3189	DB01879,DB02435,DB02995,DB03089,DB03397,DB04174,DB04474,
2.7.7.23	R_UAGDP	yes	yes	no	b3730	DB02196,DB03397,DB03814,
	R_UAGPT3	yes	yes	yes	b0090	-
6.3.2.9	R_UAMAGS	yes	yes	no	b0088	-
6.3.2.8	R_UAMAS	yes	yes	no	b0091	-
1.1.1.158	R_UAPGR	yes	yes	yes	b3972	-
	R_UDCPDPS	yes	yes	no	b0174	-
5.1.3.2	R_UDPG4E	no	no	no	b0759	DB01861,DB02196,DB02421,DB02790,DB03095,DB03397,DB04097,DB04355,
5.4.99.9	R_UDPGALM	no	no	no	b2036	DB03709,
3.6.1.45	R_UDPGALPpp	no	no	no	b0480	-
1.1.1.22	R_UDPGD	no	no	no	b2028	DB01713,
	R_UDPGDC	no	no	no	b2255	-
3.6.1.45	R_UDPGPpp	no	no	no	b0480	-
	R_UDPKAAT	no	no	no	b2253	-
3.6.1.45	R_UGLCURPpp	no	no	no	b0480	-
2.7.7.12	R_UGLT	no	no	no	b0758	DB01861,
3.5.3.19	R_UGLYCH	no	no	no	b0505	-
6.3.2.15	R_UGMDDS	yes	yes	yes	b0086	-
	R_UHGADA	yes	yes	yes	b0096	-
	R_ULA4NFT	no	no	no	b2255	-
	R_UM3PL	no	no	no	b4233	-
	R_UM4PCP	no	no	no	b1192	-
	R_UM4PL	no	no	no	b4233	-
	R_UPLA4FNT	no	no	no	b2254	-
4.2.1.75	R_UPP3S	yes	yes	no	b3804	-
4.1.1.37	R_UPPDC1	no	yes	yes	b3997	-
2.4.2.9	R_UPPRT	no	no	no	b2498	DB01632,
	R_URAt2pp	no	no	no	b2497	-
	R_URAt2rpp	no	no	no	b1006	-
1.1.1.154	R_URDGLYCD	no	no	no	b0517	-
	R_UREAtpp	no	no	no	b3927	-
	R_URIDK2r	yes	no	no	b0171	-
2.7.1.48	R_URIK2	no	no	no	b2066	-
	R_URIt2rpp	no	no	no	b2406	-
	R_URItex	no	no	no	b0411	-
	R_USHD	yes	yes	yes	b0524	-
	R_VALabcpp	no	no	no	(b3454 and b3455 and b3457 and b3460 and b3456)	-
	R_VALt2rpp	no	no	no	b0401	-
2.6.1.42	R_VALTA	no	no	no	b3770	DB01813,DB02142,DB02635,DB03993,DB04063,
6.1.1.9	R_VALTRS	yes	no	yes	b4258	-
2.6.1.66	R_VPAMT	no	no	no	b3572	-
	R_X5PL3E	no	no	no	b4197	-
1.1.1.204	R_XAND	no	no	no	(b2866 and b2867 and b2868)	-
	R_XANt2pp	no	no	no	b3654	-
2.4.2.22	R_XPPT	no	no	no	b0238	DB03942,
3.2.2.8	R_XTSNH	no	no	no	b0030	-
	R_XTSNt2rpp	no	no	no	b2406	-
	R_XYLabcpp	no	no	no	(b3566 and b3567 and b3568)	-

5.3.1.5	R_XYLI1	no	no	no	b3565	DB01881,DB01914,DB02172,DB02438,DB03206,DB03564,DB03911,
5.3.1.5	R_XYLI2	no	no	no	b3565	DB01881,DB01914,DB02172,DB02438,DB03206,DB03564,DB03911,
	R_XYLt2pp	no	no	no	b4031	-
	R_XYLUt2pp	no	no	no	(b3577 and b3578 and b3579)	-
	R_ZN2abcpp	no	no	no	b3469	-
	R_ZN2tpp	no	no	no	b3040	-
	R_ZNabcpp	no	no	no	(b1857 and b1859 and b1858)	-
